# Experimental Evolution of *Campylobacter jejuni* Leads to Loss of Motility, *rpo*N (σ54) Deletion and Genome Reduction

**DOI:** 10.3389/fmicb.2020.579989

**Published:** 2020-11-06

**Authors:** Azam A. Sher, John P. Jerome, Julia A. Bell, Julian Yu, Hahyung Y. Kim, Jeffrey E. Barrick, Linda S. Mansfield

**Affiliations:** ^1^Comparative Enteric Diseases Laboratory, East Lansing, MI, United States; ^2^Comparative Medicine and Integrative Biology, College of Veterinary Medicine, Michigan State University, East Lansing, MI, United States; ^3^BEACON Center for the Study of Evolution in Action, Michigan State University, East Lansing, MI, United States; ^4^Department of Microbiology and Molecular Genetics, Michigan State University, East Lansing, MI, United States; ^5^Department of Molecular Biosciences, The University of Texas at Austin, Austin, TX, United States

**Keywords:** *Campylobacter jejuni*, laboratory evolution, phase variation, motility, *Rpo*N

## Abstract

Evolution experiments in the laboratory have focused heavily on model organisms, often to the exclusion of clinically relevant pathogens. The foodborne bacterial pathogen *Campylobacter jejuni* belongs to a genus whose genomes are small compared to those of its closest genomic relative, the free-living genus *Sulfurospirillum*, suggesting genome reduction during the course of evolution to host association. In an *in vitro* experiment, *C. jejuni* serially passaged in rich medium in the laboratory exhibited loss of flagellar motility–an essential function for host colonization. At early time points the motility defect was often reversible, but after 35 days of serial culture, motility was irreversibly lost in most cells in 5 independently evolved populations. Population re-sequencing revealed disruptive mutations to genes in the flagellar transcriptional cascade, *rpo*N (σ54)—therefore disrupting the expression of the genes σ54 regulates—coupled with deletion of *rpo*N in all evolved lines. Additional mutations were detected in virulence-related loci. In separate *in vivo* experiments, we demonstrate that a phase variable (reversible) motility mutant carrying an adenine deletion within a homopolymeric tract resulting in truncation of the flagellar biosynthesis gene *fli*R was deficient for colonization in a C57BL/6 IL-10^–/–^ mouse disease model. Re-insertion of an adenine residue partially restored motility and ability to colonize mice. Thus, a pathogenic *C. jejuni* strain was rapidly attenuated by experimental laboratory evolution and demonstrated genomic instability during this evolutionary process. The changes observed suggest *C. jejuni* is able to evolve in a novel environment through genome reduction as well as transition, transversion, and slip-strand mutations.

## Introduction

The natural reservoir of the bacterial pathogen *Campylobacter jejuni* is avian species (Butzler, 2004; Young et al., 2007). In birds, this infection rarely results in disease and colonization is stable over long time periods (Young et al., 2007). However, *C. jejuni* colonizes numerous other animals, including humans (Butzler, 2004; Young et al., 2007). The Centers for Disease Control and Prevention (CDC) estimate that *Campylobacter* causes over 1.3 million human illnesses every year in the United States, and *C. jejuni* is the major cause of these infections (CDC, 2017). *C. jejuni* was documented as the third leading cause of human infections and the second leading cause of hospitalizations due to a foodborne bacterium (Scallan et al., 2011). *C. jejuni* infection in humans usually results in self-limiting gastroenteritis, but chronic autoimmune sequelae may occur including inflammatory bowel disease (Gradel et al., 2009) and the neurological disease Guillain-Barré syndrome (Butzler, 2004). *C. jejuni* has the potential to adapt, colonize and survive in these different niches because its genome is susceptible to genetic changes via multiple mechanisms including recombination and phase variation of contingency genes that have highly mutable homopolymeric tracts (Wilson et al., 2009; Jerome et al., 2011; Thomas et al., 2014).

Relatively little is known about the molecular factors essential for *C. jejuni* pathogenesis, but it is widely accepted that after ingestion flagellar motility is necessary for efficient colonization of a mammalian gastrointestinal (GI) tract (Newell et al., 1985; Black et al., 1988; Jones et al., 2004; Malik-Kale et al., 2007). Motility is needed for navigation through the gastrointestinal lumen and mucus layer to enable *C. jejuni* to colonize and invade the underlying epithelial cell monolayer. Non-motile mutants are often attenuated in their interaction with epithelial cells in culture (Wassenaar et al., 1991; Grant et al., 1993) and are incapable of secreting the Cia proteins necessary for maximal invasion of epithelial cells (Konkel et al., 2004). Construction of a functional flagellum is a complex process involving numerous genes and an elaborate transcriptional cascade. Sigma factor 70 controls biosynthesis of the flagellar export apparatus, FlhF, and *Rpo*N (σ54). Current models of *C. jejuni* flagellar biosynthesis show that the type III flagellar secretion system and its assembly with the inner membrane anchor and motor protein complexes, together with FlhF, influence the activity of the FlgSR two-component signal transduction pathway; FlgR in turn is required to stimulate activity of the alternative sigma factor, σ54 (*Rpo*N) (Gilbreath et al., 2011; Burnham et al., 2020). The σ54 regulon is almost exclusively composed of flagellar structural genes in *C. jejuni*, and expression of these genes generates essential components of flagella, including the basal body, hook and minor flagellin subunits (Chaudhuri et al., 2011). Researchers have also shown that *C. jejuni* flagellin must be extensively glycosylated for filament polymerization, and thus some genes involved in flagellar glycosylation are necessary for motility (Goon et al., 2003). Finally, a number of other gene products have been implicated in flagellar motility, but their molecular roles in the process are unknown. These include a predicted transmembrane protein *Cj*0390 (Colegio et al., 2001; Coward et al., 2006), the GTPase FlhF, that contributes to the activity of σ54 via FlgR/FlgS (Balaban et al., 2009; Li et al., 2020), and a protein that is potentially secreted from the cytoplasm, PflA (Yao et al., 1994).

Researchers have also described reversible, or phase variable, expression of flagellar motility in *C. jejuni* (Caldwell et al., 1985; Nuijten et al., 1989; Karlyshev et al., 2002; Hendrixson, 2006, 2008). It has been noted that the rate of motility phase switching from ON to OFF is significantly higher than from OFF to ON during *in vitro* culture (Caldwell et al., 1985). Moreover, mutants defective in flagellar biosynthesis have been reported to have an increased growth rate in culture (Wösten et al., 2004). In contrast, motile phase ON mutants seem to have higher fitness *in vivo* since non-motile cells are completely lost during gut passage when ON and OFF spontaneous mutants are co-inoculated into rabbits (Caldwell et al., 1985), humans (Black et al., 1988) or mice (Jerome and Mansfield, unpublished data). Furthermore, phase OFF non-motile isolates are reported to have low efficiencies for colonizing chickens and infant mice (Newell et al., 1985; Carrillo et al., 2004; Gaynor et al., 2004; Hendrixson, 2008). *C. jejuni* also adapts in the GI tract of chickens during the course of infection from low to high levels of colonization (Cawthraw et al., 1996; Jones et al., 2004), and the increased colonization phenotype is associated with increased motility (Jones et al., 2004).

In most work concerning the reversible motility phenotype in *C. jejuni*, the genetic basis of the motility deficiency has not been investigated. However, Hendrixson has described phase variable expression through homopolymeric adenine (and/or thymine) tract slipstrand mutation of both the histidine kinase and response regulator of the FlgSR two-component system in *C. jejuni* (Hendrixson, 2006, 2008). Spontaneous mutations in *flg*S also occurred by nucleotide deletions within a short heteropolymeric repeat (Hendrixson, 2008). When motile revertants from the frameshifted *flg*S mutants were selected *in vitro*, intragenic indels ranging from 1 to 368 nucleotides were discovered that resulted in restoration of a functional open reading frame (Hendrixson, 2008). Conversely, during chicken colonization a *flg*S mutant reverted to the motile phenotype through an extragenic single nucleotide substitution in *flg*R that suppressed the *flg*S mutant phenotype. Genetically reversible motility expression may also be affected by slipstrand mutations in the motility accessory factor genes, *maf1* and *maf7*, that contain hypervariable homopolymeric tracts of guanine residues (Karlyshev et al., 2002). Also, in the closely related species *Campylobacter coli*, reversible flagellar expression has been linked to slipstrand mutation in a homopolymeric tract of thymine residues in the *flhA* gene that is part of the flagellar T3SS (Park et al., 2000). It is known that *C. jejuni* lacks many DNA repair systems (Parkhill et al., 2000; Kang and Blaser, 2006), and taken together with the findings described here, researchers anecdotally suggest that the *C. jejuni* genome is highly plastic.

Genome reduction is considered a dominant mode of bacterial evolution where non-essential genes are lost when the organism is adapting to a novel environment (Wolf and Koonin, 2013). This evolutionary phenomenon is considered a way to evade host immune responses, eliminate redundant genes and metabolic pathways, increase virulence, and permit faster growth (Weinert and Welch, 2017). Genome reduction has been described in diverse groups of bacteria, especially in intra-cellular pathogens and symbiotic organisms such as *Lactobacillus* spp., *Salmonella typhi*, *Rickettsia, Mycobacterium tuberculosis, Shigella flexneri, and Yersinia pestis* (Maurelli et al., 1998; Wolf and Koonin, 2013; Weinert and Welch, 2017). One study has reported gene gain and gene decay in the ST403 clonal complex of *C. jejuni* by comparing isolates from different hosts and environments (Morley et al., 2015). Additionally, a recent study showed that multiple genes associated with metabolic, structural and virulence functions were reduced in *Campylobacter hepaticus* (Petrovska et al., 2017). These studies were performed on isolates collected from different origins and compared with known strains. However, the mechanism and experimental evolution of genome reduction in *Campylobacter* species has not been well studied.

To investigate these phenomena further, we took two different approaches, The first approach was an *in vitro* experimental evolution study in which mouse-adapted, fully motile derivatives of *C. jejuni* ATCC700819 (NCTC11168), referred to as *C. jejuni* 11168 in this manuscript, were subjected to serial passage in rich medium, followed by population sequencing and characterization of all recovered mutants. The second approach was an *in vivo* mouse infection study using a spontaneous revertable mutant of the *fli*R gene in non-mouse-adapted *C. jejuni* 11168 to examine its colonization deficiency and reversion to motility *in vivo*.

To begin to define how *C. jejuni* can adapt genetically to different environments, we have taken a simple approach, to evolve *C. jejuni* experimentally *in vitro* in a given novel environment and then sequence the genomes of the evolved and ancestral variants. Using this approach, we previously showed that serial passage of *C. jejuni* 11168 through a mouse model of campylobacteriosis resulted in significant changes in allele frequency in hypermutable homopolymeric guanine and cytosine tracts termed contingency loci (Jerome et al., 2011). Revez et al. (2013) demonstrated similar homopolymeric tract variation in *C. jejuni* 11168 during human infection.

In the present study we assessed the genetic basis of *C. jejuni* 11168 adaptation during experimental evolution in a more controlled environment–rich broth medium in the laboratory. We hypothesized that relieving the selective pressure of the host environment would result in the loss of functions required for *C. jejuni* growth *in vivo*. We also predicted that the observed rate and genetic patterns of laboratory adaptation would lead to a better understanding of *C. jejuni* evolvability. This work shows that experimental laboratory adaptation of *C. jejuni* results in the rapid loss of flagellar motility, which is an important colonization determinant of this pathogen. We further characterized the motility loss as reversible or irreversible based on the ability or inability to restore motility by selecting for motile cells in a semi-solid agar environment. Sequencing the ancestral and evolved populations defined multiple mutations in evolved populations that are known to disrupt flagellar motility. Many of these mutations are predicted to disrupt a protein coding sequence by a phase variable (reversible) mechanism driven by slipstrand mutations in homopolymeric DNA. However, the evolved lines also contained mutations in essential flagellar motility genes that are not genetically reversible, such as large genome deletions. An evolved population in which one of these large deletions predominated exhibited an increased growth rate compared to the ancestral population. In addition, mutations were detected in several genes involved in *C. jejuni* colonization and virulence.

Finally, in separate *in vivo* experiments, one reversibly non-motile isolate from a stock culture of *C. jejuni* 11168 was found to be impaired in colonization in the C57BL/6 IL-10^–/–^ mouse model of campylobacteriosis. The genotypic and phenotypic patterns of adaptive evolution defined in this work will likely apply to other highly mutable bacteria such as pathogenic *Helicobacter* species.

## Materials and Methods

### Bacteria

*Campylobacter jejuni* 11168 was used for the evolution experiments described in this study. The frozen stock from which all strains were derived was originally obtained from the American Type Culture Collection (ATCC 700819) and had been passaged on agar plates fewer than 4 times before being stored frozen at –80°C in 15% glycerol/tryptic soy broth (TSB). Mouse-adapted derivatives of this culture described in Bell et al. (2009) were used in the *in vitro* experiment, while non-mouse-adapted derivatives of the original freezer stock were isolated as described below used in the *in vivo* experiments. Unless otherwise noted, *C. jejuni* cultures were grown from freezer stocks by re-streaking onto Bolton (Oxoid) agar plates and incubation for 48–72 h at 37°C in gas exchange jars in a microaerobic environment generated by atmosphere evacuation followed by equilibration with a gas mixture of 80% N_2_, 10% CO_2_, and 10% H_2_.

Strain 11168mot- was isolated as a single colony from an NCTC11168 freezer stock and was subsequently found to be deficient in spreading by the semi-solid agar motility assay described below. To generate 11168mot+, cells from a primary streak of 11168mot- were used to seed the center of a semi-solid Bolton agar plate. Plates were incubated and monitored at 48, 72 and 96 h. After 96 h, a sterile inoculating loop was used to touch the outermost ring of spreading cells (black arrow in Figure 8A) before streaking onto a fresh Bolton agar plate. The new plate was incubated for 48 h before cells were harvested and stored as a freezer stock. We then analyzed this culture by the semi-solid agar motility assay to confirm an increased spreading phenotype and designated it 11168mot+.

### Methods Used in Both *in vitro* and *in vivo* Experiments

#### Semi-Solid Agar Motility Assays

To determine the motility of an entire *C. jejuni* population within a freezer culture, *C. jejuni* cells were streaked onto Bolton agar plates and grown for 48 h. The flat end of an autoclaved wooden applicator (Puritan^®^, Guilford, Maine) was then pressed onto *C. jejuni* cells in the primary streak and transferred to semi-solid (0.4% agar) Bolton agar plates and incubated at 37°C, 10% CO_2_. The degree of spreading was monitored at 24 and 48 h. Images were recorded after 48 h.

To characterize the strains used in the *in vivo* experiments, 11168wt, 11168mot-, and 11168mot+ we used these images to measure the amount of spread using ImageJ software (Abràmoff et al., 2004). Since spreading occurred concentrically during growth up to 48 h, we used ImageJ to measure the distance from the point of inoculation to the edge of the spread (the radius). This distance was normalized to the spreading distance of the positive control (11168wt from primary streak) on each plate. The normalized data from 11168mot- and 11168mot+ were compared to each other by a two-sample *t*-test, or to a hypothetical mean of 1 (the normalized distance of the positive control) by single sample *t*-tests and were considered significant at *P* < 0.0001. Finally, for strains 11168wt, 11168mot-, and 11168mot+ we spotted 20 single colonies onto semi-solid agar plates. Two colonies per variant were assayed for their ability to spread on the medium per semi-solid agar plate. Primary streaks of 11168wt and 11168mot- variant were also inoculated on each plate as positive and negative controls, respectively. A colony was considered motile if the degree (radius) of spreading at 48 h was greater than the non-spreading 11168mot- population that grew on the semi-solid agar but did not spread outward by flagellar motility.

#### Pour-Plate Motility Assays

To observe motility phenotypes of each evolved line during the time course of the broth evolution experiment we performed pour-plate assays as described by Caldwell et al. (1985), with a few modifications. For our assays we used Bolton medium with 0.33% agar cooled to 42°C. *C. jejuni* cells were then added to the liquid agar solution, which was then poured over base agar and allowed to solidify and dry for 2–3 h at room temperature. We aimed to dilute each sample to achieve a plate count of approximately 10–100 CFUs per plate, with 2 or 3 plates cultured per sample. These plates were incubated at 37°C in a 10% CO_2_, non-humidified incubator for 48 h or less to differentiate motile and non-motile CFUs. All non-motile CFUs were counted and marked on the plate, before extended incubation for a further 24–72 h to determine the number of initially non-motile CFUs that could revert to the motile form. Motile CFUs never formed a dense colony of growth, but rather a large, diffuse zone of spreading in the pour-plate by 48 h, while non-motile CFUs formed a dense, orange colony within the medium by 48 h. Reversibly non-motile colonies were characterized by the appearance after extended incubation of a diffuse spreading originating from the dense, orange colony. CFUs of the three mutually exclusive phenotypes: motile, reversibly non-motile and irreversibly non-motile, were counted. As expected, all CFUs in the 11168mot- population were reversibly non-motile by this assay. Examples of each CFU type are shown in Figure 3A. Fisher’s exact test was used to determine statistically significant differences in the frequency of motile and non-motile CFUs between days 0 and 5 or days 0 and 35. This test was also used to show significant frequency differences of reversibly and irreversibly non-motile CFUs between days 5 and 35 or 10 and 35. *P*-values less than 0.05 were considered significant.

#### Genome Sequencing and Analysis

For genome sequencing, samples were re-streaked from frozen cultures of day 0 and day 35 of each experimental population onto tryptic soy agar (TSA) supplemented with 5% defibrinated sheep’s blood. The entirety of the streak plate was resuspended in tryptic soy broth (TSB) and pelleted before DNA extraction using the QIAGEN DNeasy Blood and Tissue kit per the manufacturer’s instructions. Sequencing was performed at the Michigan State University Research Technology Support Facility (MSU RTSF) on an Illumina Genome Analyzer IIx according to the manufacturer’s instruction. Briefly, libraries of each sample were prepared with the Illumina TruSeq kit. Twelve samples including ten from this study were given unique multiplex ID tags (barcodes) and pooled for sequencing in one lane. Reads that passed filtering were sorted according to their respective barcode sequence for analysis. Read files were deposited at NCBI in the Sequence Read Archive (Submission Accession Number SRA049039; Study Accession Number SRP011023). Overall, the sequencing depth for each of the population sample varies from a minimum of 5 million reads to a maximum of 9 million reads. Similarly, based on the *C. jejuni* 11168 reference genome size (1.64 Mbp), the genomic coverage for each population sample ranges from a minimum of 231-fold to a maximum of 423-fold. Both statistical parameters of next-generation sequencing confirm the required sequencing robustness to perform subsequent analyses (Table 1). Coverage graphs across the reference genome for each of the five evolved populations, our stock culture of C. jejuni 11168, the C. jejuni 11168 fliR non-motile (mot-) mutant, and the C. jejuni 11168 motile revertant (mot +) are shown in Supplementary Figure 1. The *breseq* pipeline was used to predict consensus mutations present in all individuals in a population and mutations present in only a certain percentage of individuals in a sample. The *breseq* pipeline is described in documentation included with the freely available source code^1^. The *C. jejuni* 11168 genome sequence (RefSeq:NC_002163.1) was used as the reference for mapping reads. Consensus mutation predictions were essentially as described previously (Barrick et al., 2009). Mutations predicted included new junctions from split-read matches, which can define the endpoints of deletions or locations of new mobile element insertions, and predictions of large deletions from regions lacking read coverage. Point mutations present at intermediate frequencies in the population were predicted according to a procedure described previously (Barrick and Lenski, 2009) but with relaxed parameters that required only 90% of the length of a read to match the reference genome for inclusion in the analysis. Percentages of large deletions, such as those reported for *rpo*N in each population, were estimated by counting reads supporting the unique sequence junction formed by the deletion versus the number of reads supporting the mutually exclusive junction in the ancestral genome sequence indicating no deletion, as in a study of mobile-element insertions in mixed *E. coli* populations (Nahku et al., 2011). Overall read coverage across the *rpo*N gene region was checked to be sure it was consistent with deletion frequencies predicted by this procedure. A subset of mutations (*flg*S, *flg*R, *rpo*N, *Cj*0390) predicted from the *breseq* pipeline were assayed with an alternative method to validate their presence in the population. To assay for large genome deletions of the *rpo*N gene region, the Roche Expand Long Template PCR system and/or the Invitrogen Platinum^®^ *Taq* DNA Polymerase PCR system was used according to the manufacturer’s instructions to amplify across the putatively missing regions of DNA. The presence of PCR products associated with deletions (or lack of deletions) predicted by *breseq* was visualized by agarose (0.8% or 1.5%) gel electrophoresis. DNA from the ancestral *C. jejuni* populations was used as a control. To confirm small insertion or deletion mutations predicted to be present at relatively low frequency in the evolved populations, the Invitrogen Platinum *Taq* DNA Polymerase PCR system followed by Sanger sequencing at the MSU RTSF was used. From the sequencing chromatograms, a minor set of shifted peaks after the predicted indel that corresponded to the length of the indel was considered positive confirmation of the *breseq* prediction. DNA from the ancestral *C. jejuni* populations was used as a negative control.

**TABLE 1:** Sequencing coverage of the five evolved populations and the three *C. jejuni* genomes.

Accession number/sample identification	Number of sequence reads (paired-end)	Number of sequenced bases	Reference genome size	Coverage number of sequenced bases/numbers of reference bases
SRR437928.fastq Evolved Population 1	7,860,890	589.6M bases	1,641,481 bp	359×
SRR437929.fastq Evolved Population 2	6,847,704	513.6M bases	1,641,481 bp	313×
SRR437930.fastq Evolved Population 3	5,679,528	426M bases	1,641,481 bp	259×
SRR437931.fastq Evolved Population 4	9,257,382	694.3M bases	1,641,481 bp	423×
SRR437932.fastq Evolved Population 5	7,147,682	536.1M bases	1,641,481 bp	326×
SRR437922.fastq 11168wt	8,981,638	673.6M bases	1,641,481 bp	410×
SRR437923.fastq 11168mot- mutant	5,059,962	379.5M bases	1,641,481 bp	231×
SRR437924.fastq 11168mot+ revertant	5,707,248	428.0M bases	1,641,481 bp	260×

### Methods Used for *in vitro* Study Only

#### Experimental Evolution in Broth Medium

**FIGURE 1:**
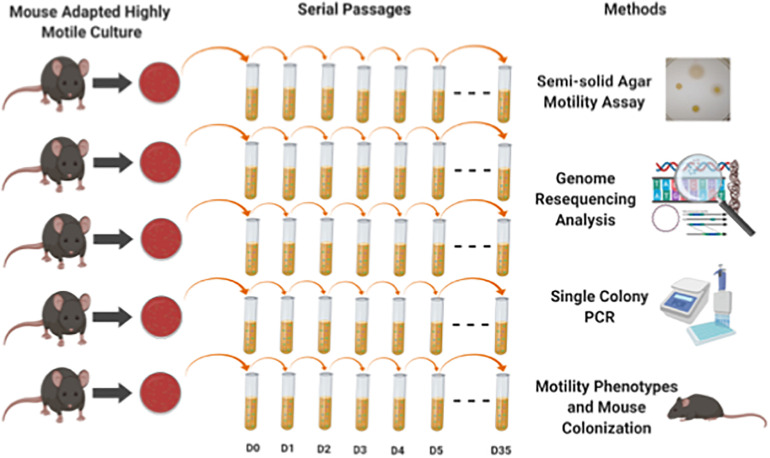
Experimental overview shows the experiment design and methods used in this study. We grew *C. jejuni* from cultures re-isolated from 5 individual mice that had been through two passages of mouse infection. These 5 non-clonal lines were used to seed five 25 cm^2^ flasks containing 10 ml autoclaved/filter sterilized Bolton broth with approximately 1 × 10^8^ cells per flask incubated under microaerobic environment. Every 24 h 100 μl of culture was transferred to a flask containing 9.9 ml fresh media (1:100 dilution). The 5 lines were kept as independently evolving populations for 35 days and cultures were saved at days 1–5, 10, 15, 25, and 35 at – 80°C in 15% glycerol/TSB. Different methods (motility assay, genome resequencing, colony PCR and mouse colonization) were used to assess both phenotypic and genomics changes in the 5 independent evolved populations.

From a previously published experiment from our lab (Bell et al., 2009), we had saved host-adapted *C. jejuni* 11168 cultures that had been passaged through the C57BL/6 IL-10-/- mouse model. Mouse passage was found to alter only the frequency of alleles present in contingency loci in these populations (Jerome et al., 2011). We grew *C. jejuni* from cultures re-isolated from 5 individual mice after two passages of mouse infection. An overview of the experiment has been shown in Figure 1. These 5 non-clonal lines were used to seed five 25 cm^2^ flasks containing 10 ml autoclaved/filter sterilized Bolton broth with approximately 1 × 10^8^ cells per flask. Flasks were placed in a gas exchange jar in a microaerobic environment generated by atmosphere evacuation and equilibration with a gas mixture of 80% N_2_, 10% CO_2_, and 10% H_2_. The jar was then incubated at 37°C with 200 rpm agitation in a tabletop shaker. Every 24 h 100 μl of culture was transferred to a flask containing 9.9 ml fresh medium (1:100 dilution), as has been done in other evolution experiments (Lenski and Travisano, 1994). The 5 lines were kept as independently evolving populations for 35 days and cultures were saved at days 1–5, 10, 15, 20, 25, and 35 at – 80°C in 15% glycerol/TSB. To determine any changes in the cell density achieved and the number of doublings during 24 h of culture, samples from day 1 and day 35 freezer stocks were first grown on Bolton agar plates, re-suspended to equal OD600 densities of approximately 0.3, and grown under the experimental evolution conditions for approximately 24 h. For each time point, one hundred microliters of the 24-h culture was used to seed each of three replicate flasks containing 9.9 ml of fresh medium. Serial dilution and plating of the inocula and test cultures after 24 h of growth under the experimental evolution conditions were performed. The difference in the number of doublings at each time point were considered significant by a *P*-value less than 0.05 by Student’s *t*-test.

#### Semi-Solid Agar Motility Assays for Populations 3 and 4

We used frozen samples collected and stored from Populations 3 and 4 on the following days of the serial passage: 0, 5, 20, and 35. First, samples were thawed on ice, diluted with TSB, and spread on solid agar plates (1.5%) to produce 100–200 isolated colonies from each sample. These plates were incubated for 72 h at 37°C under 10% CO_2_, 10% H_2_, and 80% N_2_. After 72 h, single colonies from each day were picked and touched on semi-solid agar plates using sterilized toothpicks. To make semi-solid agar plates, 1.5% Bolton agar plates were top layered with 2 ml of 0.40% Bolton agar. These streaked semi-solid agar plates were incubated for at least 96 h at 37°C under 10% CO_2_, 10% H_2_, and 80% N_2_. After the incubation, all plates were observed for motility phenotyping; motile, reversibly non-motile and irreversibly non-motile as shown in Figure 3A.

#### Semi-Solid Agar Motility Assays and Colony PCR Protocol for Populations 3 and 4

We used frozen samples collected and stored on the following days of the serial passage experiment: 0, 5, 10, 20, and 35. First, samples were thawed on ice, diluted with TSB, and spread on solid agar plates (1.5%) to produce 100–200 isolated colonies from each sample. These plates were incubated for 72 h at 37^o^C under 10% CO_2_, 10% H_2_, and 80% N_2_. After 72 h, single colonies from each day were picked and streaked on semi-solid agar plates using sterilized toothpicks. These streaked plates were incubated for at least 96 h at 37°C under 10% CO_2_, 10% H_2_, and 80% N_2_. After the incubation, 90 bacterial colonies were picked and mixed in 10 μl deionized water in 96-wells PCR plate and stored at −20°C. Later, PCR was performed using cells from these stock plates to test deletions in different genes. For this PCR, 1 μl of each single-colony stock plate suspension was added to 25 μl reaction mixture in 96-well PCR plates. Each well contained 2.5 μL 10× Buffer (MgCl_2_ free), 2.5 μL MgCl_2_ (50 mM), 2.0 μL dNTPs (2.5 mM), Taq DNA polymerase 0.25 μL, 1.0 μL both forward and reverse primers (25 pM/μL) and final volume was adjusted with sterile distilled water up to 25 μL. DNA amplification was done in an Eppendorf thermocycler using an initial denaturation step at 95°C for 10 min followed by 30 cycles of amplification (denaturation at 95°C for 1 min, annealing at 55°C for 1 min, and extension at 72°C for 1.5 min), and ending with a final extension at 72°C for 5 min. The presence of PCR products associated with deletions (or lack of deletions) predicted by *breseq* was visualized by agarose (1.5%) gel electrophoresis.

We tested 90 colonies from stored samples of Populations 3 and 4 from days 0, 5, 10, 20, and 35 of passage for deletions in the *rpo*N and *Cj*0390 genes. Sequences and details of primers used are given in Table 2. Negative control reactions contained no template DNA; template in positive control reactions was DNA isolated from the bulk culture sample from day 35 of Populations 3 or 4.

**TABLE 2:** Primers used to confirm deletions in *rpo*N and *Cj*0390.

Primer	Size (in bp)	Sequence (5’–3’)	Gene bank accession no.	Target gene	Gene location (bp)	Population
*rpo*NF	672	gggctctttgcttgctagtg	AL111168.1	*rpo*N	624211	4
*rpo*NR		tcccaaagctcttgtttgct		*rpo*N		4
*rpo*N4F	223	gggctctttgcttgctagtg	AL111168.1	*rpo*N	624194	3
*rpo*N4R		ttgagccaatgcaactatgc		*rpo*N		3
*Cj*0390F	307	atttttagcagccgtagcag	AL111168.1	*Cj*0390	357769	3, 4
*Cj*0390R		gttagcgatcgcattgatga		*Cj*0390		3, 4

#### Transmission Electron Microscopy

Bacterial samples of day 0 and day 35 from Population 4 were cultured on Bolton agar plates and incubated for 72 h in gas exchange jars with a microaerobic environment generated by atmosphere evacuation followed by equilibration with a gas mixture of 80% N_2_, 10% CO_2_, and 10% H_2_. Based on a sample processing protocol from the Center for Advanced Microscopy at Michigan State University, bacterial cells grown on these plates were fixed with 2.5% glutaraldehyde solution for 1 h. Next, a drop was placed on a formvar coated grid, stained with 1% uranyl acetate and observed in a transmission electron microscope (JEOL JEM-1400 FLASH, United States) at accelerating voltage of 100 kV.

### Methods Used for *in vivo* Study Only

#### Mouse Infection Experimental Design

All mouse breeding, husbandry, randomization to different treatments, inoculation and other procedures were performed as described previously (Mansfield et al., 2007). Briefly, C57BL/6 IL-10^–/–^ mice were conventionally reared in a specific pathogen free environment at Michigan State University and tested free of known mouse pathogens. Prior to inoculation mice were transferred to the University Research Containment Facility, separated into individual sterile filter-top cages, and fed a sterile 12% fat diet (Harlan Teklad diet 7904) with sterile water *ad libitum*. Inocula were prepared and delivered orally to 8–10 week old mice as described in Mansfield et al. (2007). Equal amounts of *C. jejuni* 11168wt, 11168mot-, or 11168mot+ (approximately 1 × 10^8^ CFU) were inoculated into 5 mice per variant. For the first experiment (Experiment 1) only 11168wt and 11168mot- were compared in 5 mice each. Subsequently all three strains were screened concurrently (Experiment 2) in 5 mice each. Serial plating and counting of CFUs in cultures before and after inoculation confirmed that viability was not altered during the inoculation process. After inoculation, mice were checked each day for the presence of clinical signs indicative of enteritis. At 10 (Experiment 2) or 11 (Experiment 1) days post inoculation, mice were humanely euthanized by CO_2_ overdose and necropsied.

#### *C. jejuni* Colonization in Mouse Fecal and Cecal Samples

Feces were collected from each mouse on day 1 and day 8 post-inoculation. A fecal pellet was collected into 1.5 ml microcentrifuge tubes and kept on ice. Four hundred microliters of TSB/15% glycerol was added to fecal pellets before they were homogenized using a sterile wooden applicator (Puritan^®^) and stored at –80°C. Serial dilution and plating of fecal slurries was performed to determine colonization levels. Five dilutions, including 100 microliters of undiluted fecal suspension, were plated per sample onto Bolton agar supplemented with the antibiotics cefoperazone (25 μg/ml), vancomycin (10 μg/ml), and amphotericin B (2 μg/ml) and incubated at 37°C in a gas exchange jar containing 80% N_2_, 10% CO_2_, and 10% H_2_ for 72 h. One hundred microliters of the slurry were also placed in a pre-weighed microcentrifuge tube and dried for 72 h at 70°C. The weight of the dried fecal slurry was measured and used to normalize the amount of feces plated between samples. Results are presented as CFU per gram dried feces. Differences in means between groups were deemed statistically significant at *P* < 0.05 using the Mann Whitney test for Experiment 1 data and the Kruskal Wallis test followed by Mann Whitney pairwise comparisons with Bonferroni correction for multiple comparisons for Experiment 2 data. During necropsy the cecum was extracted and placed into a petri plate containing approximately 20 ml phosphate buffered saline. Contents in the cecum were lightly scraped away from the tissue using scalpel and forceps. A piece of cecal tissue was saved on dry ice, and then approximately 10 millimeters of cecum that included the cecal apex was placed into a microcentrifuge tube containing 400 μl sterile TSB. Tissue was ground using a cordless pellet pestle motor (Kontes, Sigma-Aldrich, St. Louis, MO) with an autoclaved microcentrifuge pestle. Grinding was performed to yield a homogenous TSB/tissue slurry, and only a small piece of white connective tissue remained. Samples were then stored on ice prior to serial plating and testing of single colony isolates for motility.

## Results: *in vitro* Study

### Motility Was Lost During Evolution in Broth

Experimental evolution of *C. jejuni* was conducted in the laboratory in broth medium with shaking. An overview of the experiment has been shown in Figure 1. The ancestral inocula used to seed broth for each population evolved line had been passaged through C57BL/6 IL-10^–/–^ mice (Bell et al., 2009). We grew *C. jejuni* from cultures re-isolated from 5 individual mice after two passages of mouse infection. When colonies were examined from each of the five separate inocula, only motile colony forming units (CFUs) were observed by the pour plate assay (*N* = 203; total CFUs for all five populations combined; three plates per population). Five independent populations of *C. jejuni* were maintained in broth medium for 35 days by transferring a 1:100 culture volume to fresh broth after every 24 h. In the initial phase of the laboratory evolution study, wet-mount microscopy was used to monitor the evolving cultures of all populations, and it appeared that a fraction of cells in each population were losing motility over time.

**FIGURE 2:**
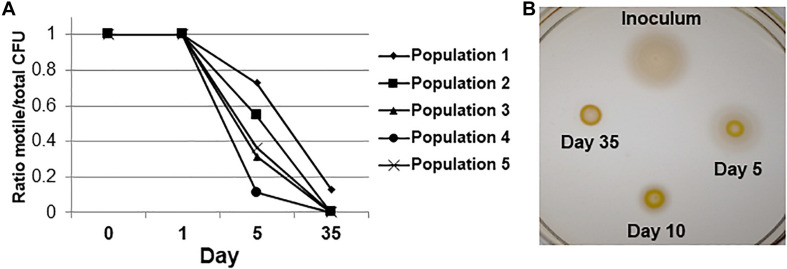
Motility loss during adaptive laboratory evolution. **(A)** Fractions of motile cells in each population over the time course of the evolution experiment by the pour plate assay. *P* < 0.05 by Fisher’s exact test for difference from day 0 between motile and non-motile CFUs for all 5 independent populations. **(B)** Semi-solid agar spreading phenotypes of a representative population over the time course of experimental evolution. The degree of spreading is representative of flagellar motility.

Some non-motile cells could be observed in the population after 3 days of serial passage by wet mount microscopy; by day 5 and thereafter there was a significant loss of motile cells in all 5 independent populations by the pour plate assay (*P* < 0.05, Fisher’s Exact test) (Figure 2A). After 35 days, 100% of observed colonies were non-spreading in 4 populations (*N* = 81, 23, 40, and 37 CFUs counted for Populations 2, 3, 4, and 5, respectively). Population 1 still contained motile CFUs after 35 days in culture, but they were present at a low frequency in the population (13%; *N* = 61 CFUs counted). A loss of motility was also evident when archived cultures were re-grown and spotted onto semi-solid agar as a population of cells at various time points (Figure 2B). However, it should be noted that when approximately 4 × 10^6^ cells from the day 35 stock cultures of all five populations were spotted at a single point on semi-solid agar and incubated for extended periods (96 h), a few spots could be observed spreading into the medium. Therefore, although motility was lost completely in 4 populations as assayed by the pour-plate method (Figure 2B), subpopulations of cells that were non-motile but had the ability to re-evolve functional flagella were still present after 35 days. Thus, the non-spreading phenotype contained both reversibly and irreversibly non-motile cells. We did additional testing to determine the proportions of each cell type found over time.

### Frequency of Irreversibly Non-motile Cells Increased During Experimental Evolution

**FIGURE 3:**
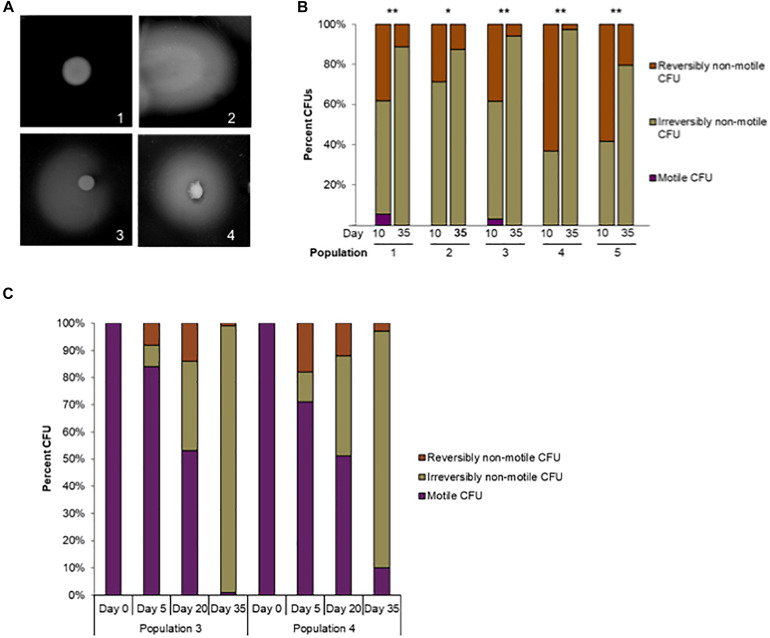
Reversible versus irreversible motility during adaptive laboratory evolution. **(A)** Phenotypes of motile, irreversibly non-motile, and reversibly non-motile CFUs in the pour-plate assay: (1) Irreversibly non-motile CFU, extended incubation (120 h); (2) motile CFU, standard assay incubation time, (48 h); (3) reversibly non-motile CFU, extended incubation (72 h); (4) reversibly non-motile CFU, extended incubation (120 h). **(B)** The percentage of reversibly non-motile, irreversibly non-motile, and motile CFUs at day 10 and day 35 were plotted for each population. ^∗^*p* < 0.01 and ^∗∗^*p* < 0.00015 by Fisher’s exact test for difference between reversibly and irreversibly non-motile CFUs at day 10 or day 35. **(C)** Reversible versus irreversible motility in Populations 3 and 4 during adaptive laboratory evolution. The percentage of reversibly non-motile, irreversibly non-motile, and motile CFUs at day 0, 5, 20 and 35 were plotted for Populations 3 and 4.

**FIGURE 4:**
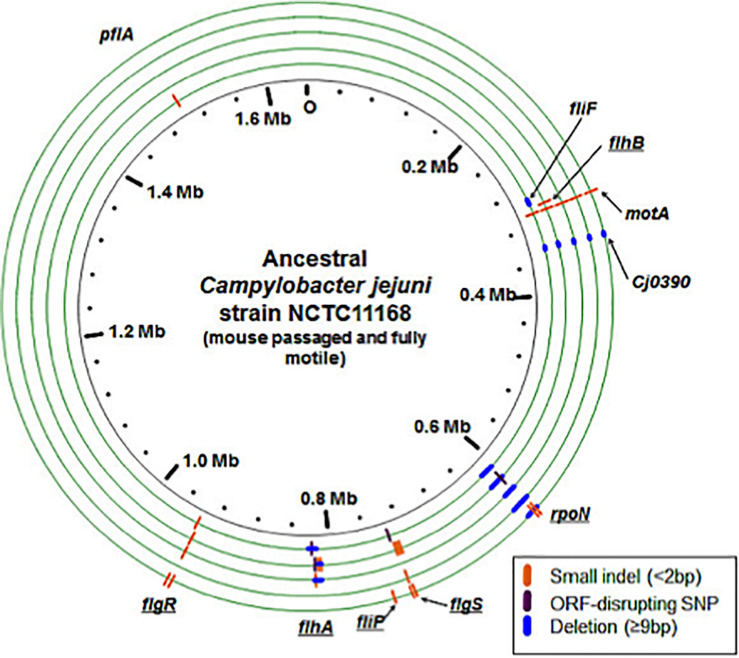
Genomic changes in evolved populations that are predicted to disrupt motility. The ancestral, mouse passaged inocula are represented by the inner black circle. Experimentally evolved lines are pictured as green circles with the innermost circle representative of Population 1 and the outermost circle representative of Population 5. Different types of mutations are indicated on each genome according to the key. Some lines were predicted to have multiple subpopulations of *rpo*N genome deletions with varying lengths, but this is not represented here: see Table 2 for details. Underlined gene names indicate genes that are predicted to decrease activity of σ54 and therefore expression of genes controlled by this sigma factor.

To further observe the motility phenotypes of individual cells in each evolving population, we used a semi-solid agar pour-plate technique (Caldwell et al., 1985) on cultures saved over the course of the evolution experiment. In this assay, non-motile cells form a small, dense, orange colony in the medium (Figure 3A-1), while motile cells spread outward to obtain more nutrients and form a diffuse circle of growth (Figure 3A-2). During the pour-plate assays described in Figure 2, we noticed that many CFUs in the populations at day 5 were able to re-evolve motility during extended incubation in the semi-solid agar environment. The restoration of motility from initially non-motile mutants in semi-solid agar during extended incubation has been demonstrated previously by Karlyshev et al. (2002) and Hendrixson (2006). Therefore, extended incubation of the pour-plates allowed us to further define non-motile cells as having reversible or irreversible phenotypes (Figures 3A,1–4).

As shown in Figure 2A, there was a significant increase in non-spreading CFUs between the time of inoculation and day 5 in all lines. Initially it was observed that of all non-motile CFUs at day 5 (*N* = 108 across all five lines), 23 spread after extended incubation, meaning that they were reversibly non-motile. At day 35 nearly all CFUs were non-motile in each population, with little reversion to a motile phenotype after 72 h. The proportion of reversibly non-motile CFUs was significantly greater at day 5 than day 35 for Populations 1, 2 and 4 by Fisher’s exact test (*P* = 0.05, < 0.0001, and 0.0006, respectively). However, highly motile CFUs from the day 5 cultures of all populations rapidly spread through the entire plate, making it difficult to accurately observe motility reversion in individual colonies during incubations longer than 72 h.

We then repeated the assay using day 10 and day 35 cultures: time points when few motile CFUs remained in each population. Nearly all observed CFUs at both times were non-motile, but a significantly higher frequency of reversibly non-motile CFUs was observed at day 10 versus day 35 for all 5 independent populations (*P* ≤ 0.01, Fisher’s exact test) (Figure 3B). The frequency of reversibly non-motile CFUs at day 10 was 3. 6-, 2. 3-, 6. 8-, 22. 9-, and 2.9-fold higher than after 35 days for Populations 1 through 5, respectively. The decrease in reversibly non-motile CFUs between day 10 and day 35 indicated that reversible motility mutations appeared early during laboratory evolution but were eventually outcompeted over the course of the experiment by *C. jejuni* cells that were irreversibly deficient for motility. To examine this phenomenon further and confirm the loss of motility over the course of the experiment, we screened an additional 100 colonies from stored samples of day 0, 5, 20 and 35 from Populations 3 and 4. A motility assay was performed to determine the phenotype (reversibly non-motile, irreversibly non-motile, or motile CFUs) of a single colony for each day mentioned above; all results are shown in Figure 3C. It was found that all 100 colonies from initial inocula of both Population 3 and 4 were fully motile. At day 5, irreversibly non-motile colonies were only 8 and 11 CFUs which increased to 33 and 37 CFUs at day 20 for Populations 3 and 4, respectively. Similarly, there was a significant loss of motility observed in each of Populations 3 and 4 at day 35 (98% and 87% for Populations 3 and 4, respectively) (Figure 3C). Unfortunately, due to multiple freezing and thawing for different assays, day 10 samples from both populations were exhausted. Overall, these findings show that inoculum of each population was initially motile, but later every passaged population started losing motility in this experiment. There were also three different motility phenotypes (reversibly non-motile, irreversibly non-motile, and motile CFUs) present in different proportions in all populations before a non-motile phenotype predominated (Figures 3B,C).

### Multiple Flagellar Motility Mutations Including Parallel *rpo*N Deletions Were Detected in Evolved Populations

We grew *C. jejuni* from cultures re-isolated from 5 individual mice after two passages of mouse infection. An overview of the experiment is shown in Figure 1. To understand the genetic basis of adaptation, we re-sequenced whole population samples of each *C. jejuni* line before and after 35 days of broth culture. Re-sequencing data from respective inocula and evolved populations were compared by mapping against the reference strain *C. jejuni* NCTC11168 genome sequence (RefSeq:NC_002163.1) for all types of mutations using the *breseq* pipeline (Table 3A). A total of 40 distinct mutations were found in 10 motility-related genes; about half of these (19, 48%) could be considered readily revertible (transitions, transversions, single base insertions and deletions) and half irrevertible (indels ≥ 2 bp). Additionally, 21 distinct mutations were found in 10 non-motility-related genes (Table 3B), of which 19 (90%) could be considered readily revertible, including presence of a duplicated 10-base non-homopolymeric tract, and 2 (10%) irrevertible.

**TABLE 3:**
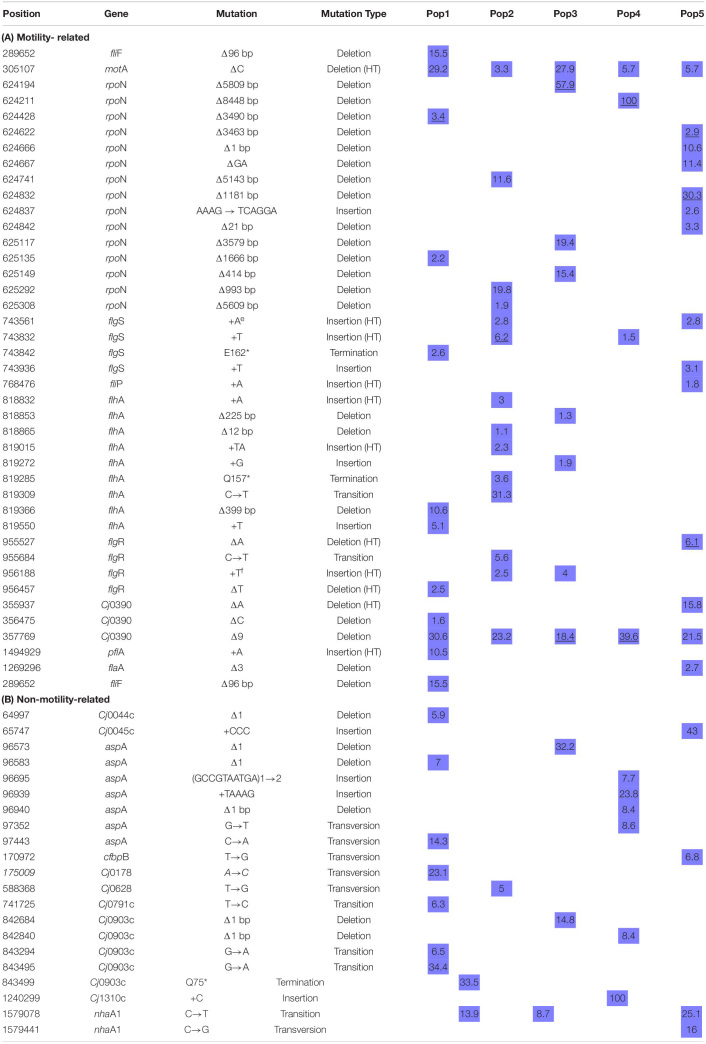
All mutations predicted in the five evolved populations.

Blue highlighted cells show presence of mutation and inside numbers are frequency (%age) of that mutation predicted by breseq pipeline.^a^+indicates an insertion of the following base(s); Δ indicates a deletion of the following base(s), or for longer deletions Δ is followed by the number of bases deleted; * symbolizes a stop codon.^b^ When the same mutation was found in multiple populations, the estimated frequencies observed for each population are listed in numerical order.(HT) indicates when a mutation occurred in a homopolymeric tract or sequence repeat of two or more units, the number of repeat units observed in the ancestral genome at the site is listed here. These mutations likely occurred by a slipstrand mechanism and are potentially reversible.^d^ Population numbers that are underlined identify mutations that were assayed and confirmed by an alternative method to the Illumina data as described in the Materials and Methods.^e^ This mutation has been reported previously by Hendrixson (2008).^f^ This mutation is in a phase variable region identified by Hendrixson (2006), but mutation in this particular thymine tract was not previously reported.

**FIGURE 5:**
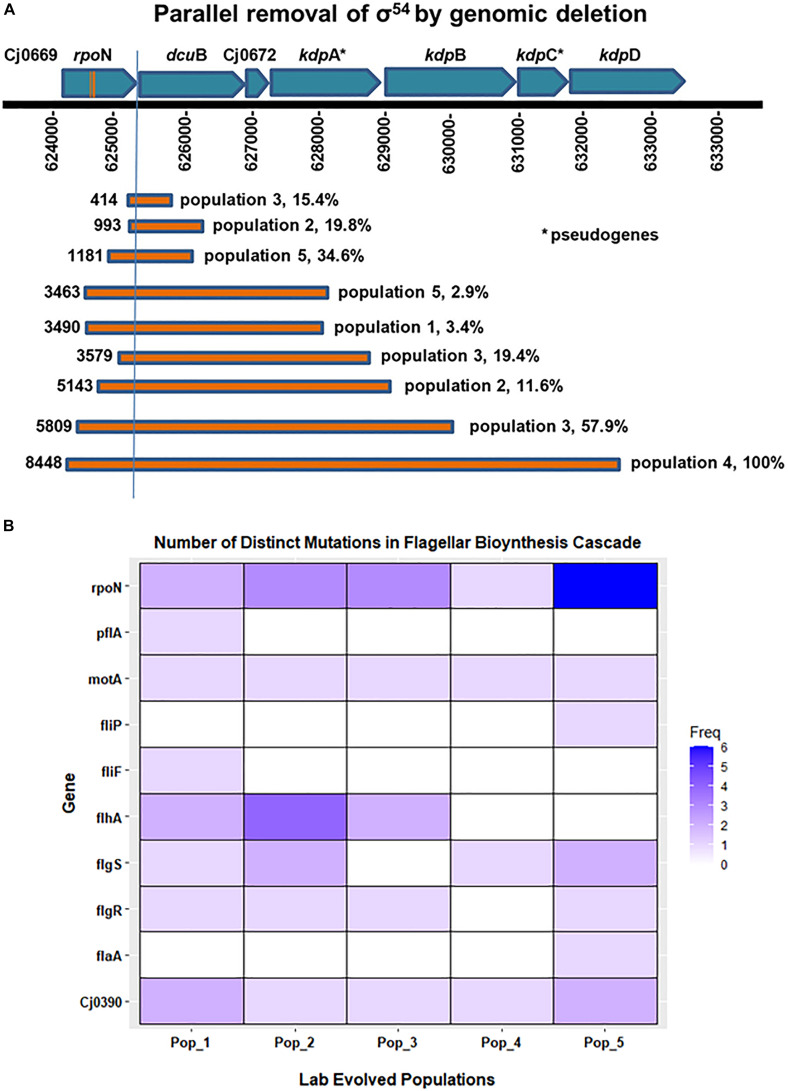
Frequency of distinct mutations in genes associated with flagellar biosynthesis cascade. **(A)** Shows parallel loss of σ^54^ by genome deletion. Bars show the different size deletions found in *C. jejuni* Populations 1–5 on Day 35 of *in vitro* growth; numbers to the left of each bar indicates the number of base pairs deleted. **(B)** The heatmap shows the presence and number of distinct mutations that occurred in genes associated with the flagellar biosynthetic cascade during the evolution of all five populations in this experiment. These distinct mutations, i.e., mutations at distinct positions with respect to the published *C. jejuni* 11168 genome, were predicted by resequencing analysis of the evolved populations.

Consistent with a loss of the motility phenotype, we discovered multiple mutations in the evolved lines that disrupt the open reading frames (ORFs) of known flagellar biosynthesis genes and genes previously shown to be involved in flagellar motility (Figure 4). There were distinct types of mutations varying from point mutations to complete gene deletions detected, including mutations in motility-related genes *flg*S, *flg*R, *flh*A, *flh*B, *fli*P, *fli*F, *mot*A, *Cj*0390, *pfl*A, and *rpo*N (Figure 5 and Table 3). For example, two distinct large deletions were detected in *rpo*N in Population 1 (3490 and 1116 bp at positions 624428 and 625135, respectively); the *flg*S locus sustained a single termination mutation at position 743842 in Population 1 and two separate single base pair insertions at positions 743561 and 743832 in Population 2.

Overall, the estimated frequencies of many of the flagellar gene mutations were low in the population by day 35 (Table 3A). However, none of these mutations were detectable in the re-sequencing data on the ancestral inocula, and a subset of these predictions was confirmed by independent methods by PCR and Sanger sequencing (Table 3A). It should be noted that synonymous and non-synonymous single nucleotide polymorphisms (SNPs) were also detected at low frequency in many of these genes after broth adaptation, but without testing the phenotypic effect of these alleles there is no clear evidence for whether they alter motility. Unless a SNP introduced a premature stop codon it is listed in Table 3 but presumably some non-synonymous SNPs, in-frame deletions, and premature stops near the 3-prime end of the protein might also disrupt gene function.

On day 35, we found ORF-disrupting mutations in the early genes of the flagellar transcription cascade, those involved in the type III secretion system (*flg*S, *flg*R, *flh*A, and *fli*P). These mutations were mainly easily revertible. Functional protein products of these genes are necessary for expression of the σ54 regulon (Hendrixson and DiRita, 2003; Joslin and Hendrixson, 2009). In addition, all 5 evolved populations contained a fraction of cells that had lost some portion of the σ54-encoding gene, *rpo*N (Table 3A). Deletions that included part of *rpo*N ranged in size from 21 to 8448 bases. One evolved population (Population 4) contained an *rpo*N mutation that approached fixation during broth evolution. This mutation was an approximately 8.5 kilobase deletion that removed most of the *rpo*N locus along with the downstream genes *dcu*B, *Cj*0672, *kdp*B, *kdp*C and 330 amino acids from the N-terminus of *kdp*D. Consistent with the irreversible motility loss described above, deletion of *rpo*N from the *C. jejuni* genome results in the loss of expression of flagellar biosynthesis genes (Hendrixson and DiRita, 2003; Chaudhuri et al., 2011), and a non-flagellated, non-motile phenotype (Jagannathan et al., 2001). To assay for a growth advantage under the experimental evolution conditions, we revived frozen day 1 and day 35 samples from Population 4 to estimate the number of doublings per 24-h growth period. On average, day 35 samples doubled significantly more (4.1 times), than day 1 samples (3.7 times) (*P* = 0.007). This suggested the deletion of *rpo*N resulted in a higher cell density after 24 h of growth when serial transfer to new medium occurred during the evolution experiment. The parallel loss of σ54 expression through mutations disrupting the flagellar transcriptional cascade or actual deletion of *rpo*N in all 5 lines, along with the evidence of a growth advantage, supports the conclusion that motility loss through loss of *rpo*N and therefore expression of its regulon was favored by selection under the experimental evolution conditions.

### ORF-Disrupting Reversible and Irreversible Types of Mutations Were Detected

Since we observed phenotypically reversible and irreversible motility phenotypes during the pour-plate assay, we hypothesized that some of the ORF-disrupting mutations discovered might be subject to phase variable expression. The disruptive types of mutations we found included single base insertion and deletion mutations–often in homopolymeric tracts of DNA; expansion of a TA dinucleotide repeat; large DNA deletions; and SNPs that introduced premature stop codons. Of these mutation types, insertions and deletions in homopolymeric DNA are considered highly mutable by slipstrand mispairing (Levinson and Gutman, 1987), and are the only documented source of phase variable gene expression in *C. jejuni*. Mutations in long tracts (7–8 base) of homopolymeric DNA were found in *flg*S, *flg*R, *flh*B, and *fli*P genes, while mutations in short homopolymeric tracts (3–5 base) that may be reversible, but presumably have a lower mutation rate, were found in *flg*S, *flg*R, *flh*A, *rpo*N, *pfl*A, and *mot*A genes. All of the homopolymeric tract indel mutations discovered were in adenine or thymine tracts, except for the mutation in *mot*A that occurred within a 5 base guanine tract and was present in all 5 evolved lines. It should be noted that some of these mutations occurred in multiple lines or have been described in past literature (Table 3), strongly supporting the conclusion that these are defined sites of increased mutability and sources of phase variation based on the instability of homopolymeric DNA in *C. jejuni*.

### Accumulation of Mutations and Parallel Loss of *rpo*N Over the Course of Experimental Evolution

From the Illumina genome resequencing data analysis, we found a pattern of genomic changes present in all population samples (Table 3) during adaptation of mouse-passaged inocula to growth in rich broth in shaking cultures under microaerophilic conditions. Large ORF-disrupting deletions occurred in the *rpo*N gene which is a crucial regulator of flagellar synthesis cascade in *Campylobacter* (Hendrixson and DiRita, 2003; Chaudhuri et al., 2011; Lertsethtakarn et al., 2011). Similarly, we found a 9 bp deletion in the *Cj*0390 gene, *pfl*B, which plays a role in motility and colonization of *C. jejuni* in chickens (Kanji et al., 2015). This 9 base pair deletion was predicted to be in-frame and was found in all evolved populations (Table 3A). We chose these two deletions for further study (1) because they arose in all five populations and (2) because they were large enough not to be revertible during growth for further study.

Based on the motility assays and genome sequencing in this study, it seemed possible that the evolution of non-motility passed through a phase in which most of the mutants were revertible before entering a phase in which non-revertible mutants predominated. We hypothesized that this transition of reversible motility to non-reversible motility occurred due to accumulation of non-revertible mutations and parallel loss of *rpo*N by deletion in all populations. To test this hypothesis, we isolated single colony-forming units (CFUs) and performed colony PCR to delineate the trajectory of the loss of *rpo*N and accumulation of *Cj*0390 mutants over the whole course of the experiment; phenotypic results are shown in Figure 3C. We examined 90 CFUs isolated from stored samples of Populations 3 and 4 from each of days 0, 5, 10, 20, and 35 and determined the presence of mutations in the *rpo*N and *Cj*0390 genes. We chose Populations 3 and 4 because of the presence of high frequencies of *rpo*N and *Cj*0390 gene deletions predicted by *breseq* analysis, and to be consistent with our previous motility assay experiment shown in Figure 3C. Next we went on to examine the genotypic results.

**FIGURE 6:**
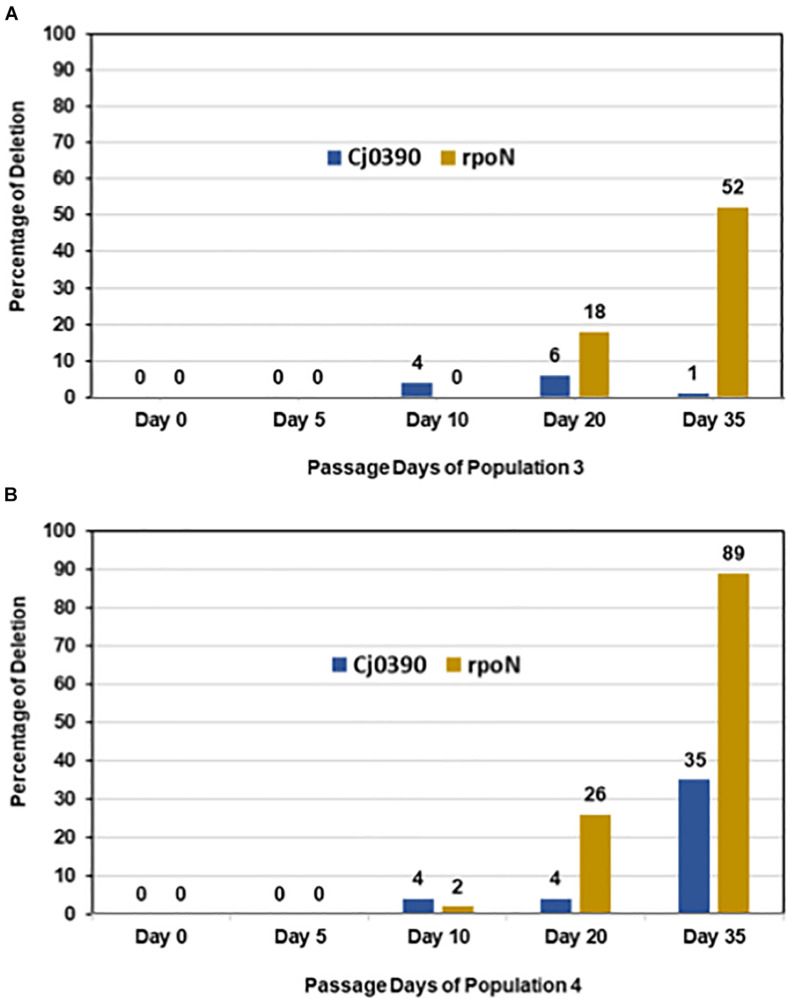
Accumulation of irreversible mutations along with loss of the *rpo*N gene. Figure shows percentage of colonies from Populations 3 and 4 having deletions in *rpo*N and *Cj*0390 detected by colony PCR on days 0, 5, 10, 20 and 35. The percentage was calculated out of 90 colonies tested by colony PCR from each day. **(A)** Population 3; **(B)** Population 4.

In colony PCR screening, it was found that no mutations in the *rpo*N gene were detected in day 0 colonies, which is consistent with the Illumina sequencing data where no deletions in *rpo*N were detected in the initial inoculum for Population 4. Similarly, there were no deletions detected in *rpo*N in all 90 colonies screened from day 5 Population 4. However, there was an increasing trend of colonies, 2%, 26% and 89%, grown from Population 4 on days 10, 20 and 35, respectively, which showed deletion of the *rpo*N gene (Figure 6B). The 89% of colonies with *rpo*N deletion mutations as detected by PCR at day 35 is consistent with the Illumina resequencing data where 100% of reads mapping to the *rpo*N gene showed an 8448 bp deletion beginning at the C terminal half of *rpo*N and extending downstream through several genes and pseudo-genes into *kdp*D (Table 3A). Next, there was no deletion present in the *Cj*0390 gene in all colonies grown from Population 4 on days 0, 5 and 10. However, 4% and 35% of colonies showed a deletion in the *Cj*0390 gene from Population 4 on days 20 and 35, respectively. This percentage of deletion mutants at day 35 is also consistent with Illumina sequencing data of the inoculum and day 35 samples (Table 3A). Thus, these percentages are repeatable and based on two sensitive methods where the limits of detection are quite low.

Similarly, colony PCR was performed to detect a 5809 bp deletion in the *rpo*N gene (starting at position 624194) and the 9 bp deletion in *Cj*0390 in Population 3 (Table 3A). It was found that the 5809 bp *rpo*N gene deletion was not present in colonies grown from days 0, 5, and 10 of Population 3. However, 18 and 52% of colonies were detected with a deletion in the *rpo*N gene in Population 3 at days 20 and 35, respectively (Figure 6A). The percentage of deletion mutants in the *rpo*N gene at day 35 detected by colony PCR was again consistent with the genome resequencing data where 57.9% variants were found with a 5809 bp deletion (*rpo*N4) in *rpo*N gene in Population 3 (Table 3A). There was no deletion found in the *Cj*0390 gene at days 0, 5, and 10 in Population 3. However, 6 and 1% deletion mutants of *Cj*0390 gene were found by colony PCR in Population 3 at days 20 and 35, respectively (Figure 6A). Thus, the findings combined from our motility assay and colony PCRs demonstrate how the early reversible loss of motility through mutations in many different genes was followed by an accumulation of non-revertible loss-of-function mutations associated with the motility regulator gene *rpo*N.

### Flagella Were Lost in Evolved Population 4 During Evolution in Broth

**FIGURE 7:**
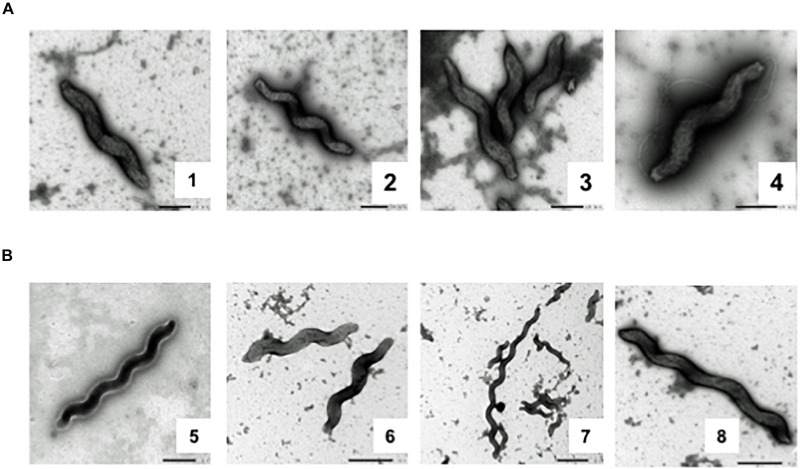
Transmission electron micrographs of Day 0 and Day 35 samples from Population 4. Electron micrographs of days 0 and 35 samples from Population 4 **(A)** Bacterial cells from day 0 have a typical bipolar flagellar morphology. **(B)** Cells from day 35 have completely lost flagella.

**FIGURE 8:**
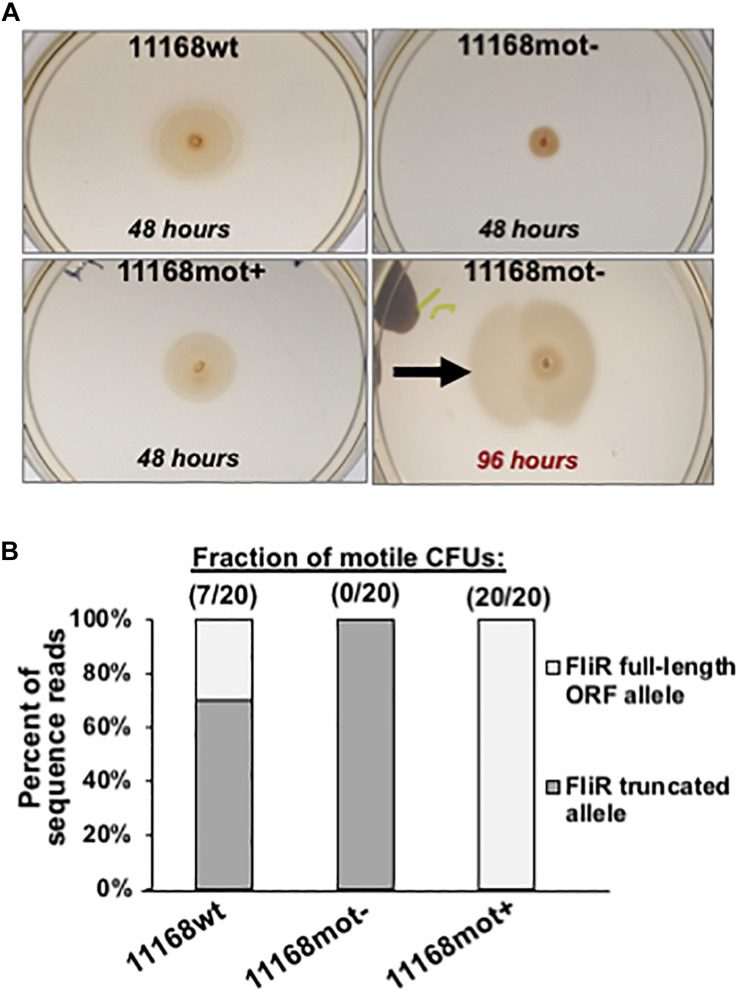
Phenotype and genotype data for 11168wt, 11168mot- and 11168mot+. **(A)** Spreading phenotypes of the 3 variants after incubation on semi-solid agar. Plates were imaged after 48 h for each variant. Also pictured is the 11168mot- variant after extended incubation (96 h) to show the selection for motile revertants. The two images of 11168mot- are from the same plate incubated for different times and the black arrow indicates the location where cells were isolated to generate 11168mot+. Variants and incubation times are listed on the figure. **(B)** The predicted frequency of 987 full-length FliR open-reading frame alleles present in each variant by the Illumina 988 sequence data is plotted. This frequency is based on the number of sequence reads with an 8 base homopolymeric adenine tract (full-length ORF), or 7 base tract (truncated ORF by frameshift mutation). The observed frequency of motile CFUs for each population is listed above each bar showing the tight association of the population level motility phenotype to *fliR* genotype.

After observing motility loss and genomic changes during the experiment, we performed transmission electron microscopy on day 0 and day 35 samples from Population 4. All bacterial cells observed from day 0 had bi-flagellar morphology (Figures 7A, 1–4). On the contrary, bacteria from day 35 were completely missing flagella (Figures 7B, 5–8). In addition, day 35 cells showed increased morphological variation, including elongated cells, as previously reported (Hwang et al., 2011; Figures 7B,5–8). These morphological findings confirm that disruption in the flagellar transcriptional cascade by loss of *rpo*N caused complete loss of the flagellar filament.

### Mutations in Non-motility-Related Loci Were Mainly Revertible

Table 3B contains the 21 non-motility-related mutations detected in the five evolved populations. Eighteen of the 21 were single base pair changes; no large deletions were detected. It is noteworthy that a number of these mutations affect loci involved in colonization and virulence: *Cj*0045c (colonization) (Kim et al., 2012; Artymovich et al., 2013), *cfbp*B and *chu*A (*Cj* 0174 and *Cj*0178; iron uptake and heme transporter, respectively) (Hofreuter et al., 2006; Ridley et al., 2006), c*ap*A (*Cj*0628; colonization and adherence to host cells) (Ashgar et al., 2007), *liv*J (*Cj*0903; colonization) (Hendrixson and DiRita, 2004), and *nha*A (*Cj*1655; colonization) (Gao et al., 2017) *Cj*1310c is part of the complex locus encoding O-linked glycosylation of the flagellar filament; the O-linked glycosylation system has been implicated in colonization and adherence to host cells (Jones et al., 2004). In addition, since the flagellum is a major antigen, mutations in this protein may contribute to immune evasion.

## Results: *in vivo* Study

### Isolation of A Spontaneous Motility Mutant and Experimental Selection of a Motile Revertant

Our evolution experiment shows that motility deficient cells are rapidly selected in *C. jejuni* during laboratory culture, and we predicted that this might lead to spontaneous motility-deficient mutants in stock cultures. Indeed, when 20 individual colonies from a wild-type *C. jejuni* ATCC 700819 stock culture were spotted onto semi-solid agar 14 of 20 colonies showed little to no spreading in semi-solid agar, indicating that this ATCC 700819 stock was mostly made up of non-motile cells.

From our stock culture a non-spreading mutant was isolated and stored. Twenty colonies from this putatively non-motile stock were assayed for spreading on semi-solid agar; no colonies spread by flagellar motility from the point of inoculation. However, when these non-motile cells were left on the semi-solid agar for an extended incubation, spreading sectors arose (Figure 8A, 96 h), indicating that the motility loss was revertible. Cells from one of these sectors were isolated and stored. When 20 colonies from this variant were assayed for motility in semi-solid agar, all 20 were relatively uniform in spreading phenotype, spread approximately 3-fold more than the non-spreading control, but based on colony diameter measurements spread on average approximately 70% as much as the wild-type culture. Thus, the motile phenotype may not have been fully restored in this revertant. However, since the number of viable cells transferred from the original colonies to the semi-solid assay plates on the end of the applicator stick could have varied between the three strains, this conclusion must remain tentative. We will refer to the original mixed-motility stock culture as 11168wt, the non-motile isolate as 11168mot-, and the motile revertant as 11168mot+.

By genome sequencing we discovered that the motility defect of 11168mot- was due to a single nucleotide deletion in an eight base homopolymeric thymine tract within the open-reading frame of *fli*R, a gene that codes for a structural protein of the flagellar type III secretion system (T3SS) in *C. jejuni* (Gilbreath et al., 2011). This mutation shifts the *fli*R open-reading frame to introduce a stop codon after 39 amino acids; a significant truncation since the full-length ORF is predicted to be 255 amino acids. No mutations in *fli*R were detected during broth experimental evolution, but as is the case with *flh*A, *flh*B, *flg*S, *flg*R and *fli*P, *fli*R disruption results in repression of the σ54 regulon (Hendrixson and DiRita, 2003). Consistent with our motility phenotype observations, this deletion was present in approximately 70% of the reads in the 11168wt culture and 100% of reads for 11168mot- (Figure 8B). Selection for motility on semi-solid agar resulted in the re-insertion of an adenine residue in this tract to restore the *fli*R ORF, and only the allele that generated the full-length ORF was present in 11168mot+ (Figure 8B).

Finally, it must be noted that, as in the results obtained by Gaynor et al. (2004) and Cooper et al. (2013), genome sequencing revealed genetic differences between the published *C. jejuni* 11168 sequence (Parkhill et al., 2000), our stock culture (11168wt), the non-motile mutant, 11168mot-, and the motile revertant 11168mot+. These differences are shown in Table 4. Thirty-two differences from the originally published *C. jejuni* 11168 sequence (Parkhill et al., 2000) were detected; 16 were found at 100% frequency in all three strains, while alleles at the other 16 loci varied in frequency among the three strains. Fourteen of the 32 loci exhibited changes in lengths of homopolymeric tracts. Among the sixteen changes that distinguish 11168mot- and/or 11168mot+ from the 11168wt parent stock are alterations in loci shown to affect motility, colonization proficiency, or human disease. These loci include *Cj*0045, *Cj*0170 (Kim et al., 2012; Artymovich et al., 2013), *cip*A (Crofts et al., 2018), *che*V (Gao et al., 2017), and three loci in the O-linked flagellar glycosylation pathway, *Cj*1295, *Cj*1318, and *Cj*1325 (Howard et al., 2009). The latter pathway has been implicated in colonization (Thibodeau et al., 2015). One or more of the changes in these loci may be responsible for the lack of full restoration of motility in 11168mot+.

**TABLE 4:** Percent of reads showing sequence differences between *C. jejuni* 11168wt stock culture, 11168mot- derivative, and 11168mot+ revertant used in mouse colonization experiments.

			Percent of reads having mutation				
Position*	**Mutation**	**Annotation**	**Stock**	**Mutant**	**Revertant**	**Gene**	**Description**
**(A) Mutations not detected in all three strains**							
16,869	C→T	E179K (GAA→AAA)	0	100	100	*rrc* ←	non-heme iron protein
65,747*	+C	coding (720/723 nt)	0	100	0	*Cj*0045c ←	iron-binding protein
65,747*	+CCC	coding (720/723 nt)	30.2	0	0	*Cj*0045c ←	iron-binding protein
67,708	Δ1 bp	intergenic (−1242/+824)	100	100	0	*Cj*0045c ←/← *mnm*A	iron-binding protein/tRNA-specific 2-thiouridylase MnmA
167,292*	+G	coding (243/246 nt)	0	100	100	*Cj*0170 →	hypothetical protein
263,831	G→A	R115C(CGT→TGT)	74.0	100	100	*che*V ←	chemotaxis protein
387,189	T→G	S8A (TCT→GCT)	0	0	100	*Cj*0423 →	integral membrane protein
527,363	A→C	intergenic (+ 8/−819)	6.9	0	9.5	*Cj*0564 →/→ *Cj*0566	integral membrane protein/hypothetical protein
550,127	G→A	G101G (GGG→GGA)	6.7	0	0	*Cj*0590 →	tRNA (cmo5U34) -methyltransferase
639,006*	+G	coding (887/1353 nt)	100	0	0	*cip*A	invasion protein CipA
1,019,216	C→G	G195A (GGC→GCC)	73.0	100	100	*Cj*1087c ←	peptidase
1,106,998*	Δ1 bp	coding (87/768 nt)	0	100	0	*fli*R ←	flagellar biosynthesis protein FliR
1,227,120*	+G	coding (143/1308 nt)	100	0	0	*Cj*1295 →	hypothetical protein
1,246,846*	Δ1 bp	coding (169/1950 nt)	100	100	0	*Cj*1318 →	hypothetical protein
1,253,669*	Δ1 bp	coding (253/255 nt)	0	100	100	*Cj*1325 →	methyltransferase
1,335,700	G→A	A149V (GCT→GTT)	7.5	0	0	*tpi*A ←	triosephosphate isomerase
1,538,838	G→A	S21N (AGC→AAC)	71.0	100	100	*prf*A →	peptide chain release factor 1
**(B) Mutations detected in all three strains**							
180,706	Δ2 bp	coding (1150−1151/1155 nt)	100	100	100	*Cj*0184c ←	serine/threonine protein phosphatase
253,191	A→G	D48G (GAT→GGT)	100	100	100	*mre*B →	rod shape-determining protein MreB
262,345	A→G	I290T (ATA→ACA)	100	100	100	*che*A ←	chemotaxis histidine kinase
393,542	T→A	*205K (TAA→AAA)	100	100	100	*Cj*0431 →	ATP/GTP-binding protein
420,550	A→G	intergenic (−124/+339)	100	100	100	*Cj*0454c ←/← *Cj*0456c	membrane protein/hypothetical protein
527,378*	Δ3 bp	intergenic (+ 23/−802)	NA**	NA**	NA**	*Cj*0564 ←/← *Cj*0566	integral membrane protein/hypothetical protein
527,389*	(G)_12__→__13_	intergenic (+ 34/−793)	100	100	100	*Cj*0564 →/→ *Cj*0566	integral membrane protein/hypothetical protein
58,368*	+G	coding (501/504 nt)	100	100	100	*Cj*0628 →	lipoprotein
695,942*	+C	intergenic (+ 4358/−482)	100	100	100	*Cj*0736 →/→ *Cjr*07	hypothetical protein/16S ribosomal RNA
760,188	A→G	K198E (AAA→GAA)	100	100	100	*Cj*0807 →	oxidoreductase
1,079,739	+CC	coding (5/549 nt)	100	100	100	*Cj*1145c ←	hypothetical protein
1,189,659	A→G	E180G (GAA→GGA)	100	100	100	*por*A →	major outer membrane protein
1,234,921*	+C	coding (589/1218 nt)	100	100	100	*Cj*1305c ←	hypothetical protein
1,338,383*	+A	intergenic (−38/−21)	100	100	100	*gap*A ←/→ *nad*D	glyceraldehyde 3-phosphate dehydrogenase/nicotinate-nucleotide adenylyl transferase
1,363,821*	+C	coding (302/927 nt)	100	100	100	*Cj*1429c ←	hypothetical protein
1,404,342	+T	intergenic (+ 365/+805)	100	100	100	*Cj*1468 →/← *cts*E	integral membrane protein/type II protein secretion system protein E

Sequence differences, frequencies, and annotations were determined by BreSeq as described in Materials and Methods. Sequencing statistics are given in Table 1.*Denotes homopolymeric tract at site**Breseq could not report frequencies for the deletion.

### 11168mot- Is Deficient for Mouse Colonization and the *in vitro* Revertant, 11168mot+, Is Partially Restored for Mouse Colonization

**FIGURE 9:**
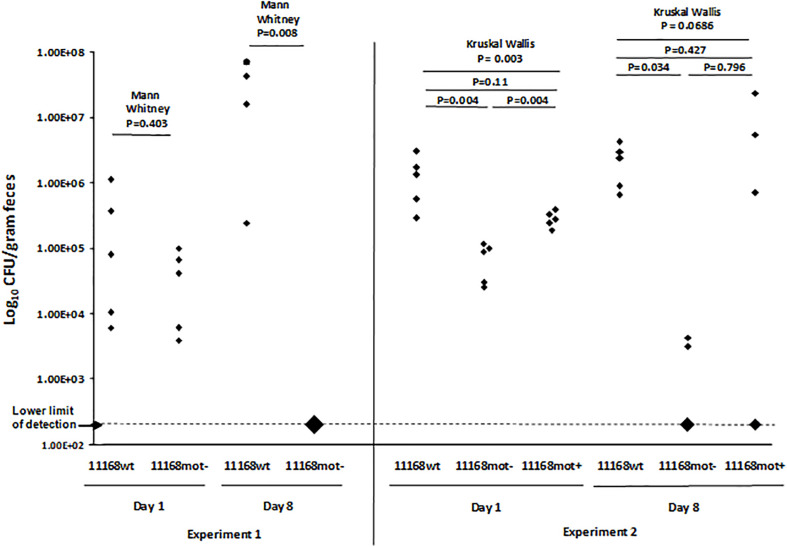
Mouse colonization of 11168wt, 11168mot-, and 11168mot+. The *C. jejuni* load in each mouse was estimated from fecal samples collected at day 1 and day 8 post-inoculation and plotted here are as log10 CFU per gram dried feces. Experiment 1 was performed with two strains, 11168wt and 11168mot-, while Experiment 2 included strains 11168wt, 11168mot- and 11168mot+. All experimental groups included 5 mice. The number of cfu inoculated per mouse were determined by serial dilution and were as follows: Experiment 1 – 11168wt, 1.9 × 10^8^; 11168mot-, 1.6 × 10^8^; Experiment 2 – 11168wt, 2.7 × 10^8^; 11168mot-, 3.3 × 10^8^; 11168mot+, 2.7 × 10^8^. All mice in which no *C. jejuni* cfu were detected are plotted on the X axis, and the size of diamond indicates the number of mice. Large diamonds for the 11168mot- group at day 8 in experiment 1 and the 11168mot- and 11168mot+ groups at day 8 in experiment 2 show 5, 3, and 2 mice, respectively. *P*-values shown above solid lines were calculated using Mann-Whitney pairwise analysis (with Bonferroni correction for multiple comparisons in Experiment 2), while *P*-values shown above dotted lines were calculated using the Kruskal Wallis test.

After defining the genotype and reversible motility phenotype of 11168wt, 11168mot-, and 11168mot+, we sought to assess the ability of these variants to colonize the C57BL/6 IL-10^–/–^ mouse model of campylobacteriosis (Mansfield et al., 2007). *C. jejuni* was detected in the feces of all mice on day 1 post infection, indicating that inoculation was successful. As can be seen in Figure 9, 11168wt was being shed in feces at a higher level than the 11168mot- variant at day 1 and day 8 post-inoculation for both independent experiments. Two mice were colonized with 11168mot- despite the initial motility defect of this variant. When *C. jejuni* from the cecal tissue of these mice were assayed by pour plate, all detectable CFUs were motile, indicating that 11168mot- had reverted to a motile form. *In vitro* reversion of 11168mot- back to the motile phase did not fully restore the ability of 11168mot- to colonize mice: only 3 of 5 mice were colonized by 11168mot+. As noted above, 11168mot- and 11168mot+ exhibit a number of genetic differences compared to 11168wt and to each other, including differences in genes affecting motility or colonization which may be responsible for the failure of 11168mot+ to colonize all inoculated mice.

## Discussion

In this work, we sought to understand how *C. jejuni* evolves outside of the host during serial transfer in laboratory medium. Anecdotal evidence has suggested that multiple passages of *C. jejuni* in the laboratory lead to attenuation in animal models. We contend that if genetically well-characterized strains are to be used to examine host interactions either *in vivo* or *in vitro*, then changes induced by laboratory passage need to be understood. Genome studies, including those of our group, suggest that *C. jejuni* may be particularly evolvable due to a lack of mutational repair pathways (Kang and Blaser, 2006), little genetic redundancy (Parkhill et al., 2000), and the presence of hypermutable homopolymeric DNA tracts (Parkhill et al., 2000; Jerome et al., 2011). Researchers have described large intragenomic rearrangements (Scott et al., 2007), horizontal genetic exchange (de Boer et al., 2002), homopolymeric adenine and thymine tract indel mutations (Hendrixson, 2006), and allele frequency changes in contingency loci (Wilson et al., 2010; Jerome et al., 2011; Bayliss et al., 2012) during *in vivo* growth of *C. jejuni*.

These findings illustrate the fact that high mutation rates and consequent homopolymeric tract variation make it particularly useful to view *C. jejuni* through the lenses of population genetics and evolution during both host infection and laboratory growth. Our analysis of the five evolved populations is explicitly directed toward this viewpoint. Furthermore, an inoculum produced for any experiment is likely to contain cells with genotypes varying at several loci. Comparison of sequence results for 11168wt, 11168mot-, and 11168mot+ illustrates this point: 11168mot- was a single colony derived from 11168wt but differs from it at several loci; likewise, 11168 mot+ was a single colony derived from 11168mot- but differs from it at several loci.

In our experiment, a *C. jejuni* strain that had been thoroughly host-adapted by multiple passages in mice was subjected to novel selective conditions in a non-host environment by *in vitro* culture in rich medium in the laboratory. We found that *C. jejuni* serially passaged in rich medium exhibited loss of flagellar motility. This was accompanied by numerous disruptive mutations to genes in the flagellar transcriptional cascade, including genes affecting the expression of *rpo*N (σ54) and the expression of the genes σ54 regulates, coupled with deletion of *rpo*N in all evolved lines. Additional mutations were also detected in virulence-related loci. Published data also support these findings. It has long been known that motility is an important host colonization determinant and that spontaneous loss of motility was possible while working with certain *Campylobacter* strains (Caldwell et al., 1985). During investigations into the role of *Rpo*N in *C. jejuni*’s defense against various stresses, Hwang et al. (2011) found that motility was quickly lost during *in vitro* growth through deletions in *rpo*N, a crucial control element in the expression both of the flagellar transcriptional cascade and several stress responses. Similarly, in studies designed to examine the genetic basis of motility loss, ORF-disrupting indel mutations in *flg*R, *flg*S and *flh*A were discovered (Park et al., 2000; Hendrixson, 2006, 2008). These mutations were found in stocks that had been cultured in the laboratory, and they disrupted downstream expression of σ54-regulated genes (Hendrixson and DiRita, 2003). In addition, Gaynor et al. (2004) described extensive virulence differences between the original clinical isolate of strain NCTC11168 (11168-O) and the genome sequenced variant (11168-GS) that included a severe motility defect in 11168-GS. Considering the pleiotropic effects of flagellar motility loss on virulence traits (Guerry, 2007), and our finding that motility deficiency is selected during laboratory culture, it is possible that the attenuated 11168-GS isolate from Gaynor’s study had acquired a motility-disrupting mutation during routine culture. This would be analogous to both the 11168mot- variant (*fli*R mutation) described in this work and the *flg*R, *flg*S, or *flh*A mutants described previously (Park et al., 2000; Hendrixson, 2006, 2008).

Loss of sigma factor function by point mutation and small indels has been observed during experimental laboratory adaptation of *E. coli* (Notley-McRobb et al., 2002; Conrad et al., 2011). It has been proposed that mutations affecting regulatory hubs such as sigma factors result in large expression changes within the cell and can therefore yield a large fitness benefit. In some cases, these mutations of large effect have been shown to increase fitness during experimental evolution and have recurrently occurred in independently evolved cultures (Cooper et al., 2003; Conrad et al., 2010). As an example, adaptive evolution experiments using *E. coli* have shown that mutations in *rpo*S, the alternative sigma factor necessary for mounting a stress response, are frequently observed in parallel cultures (Notley-McRobb et al., 2002; Ferenci, 2008). It is hypothesized that an altered balance between self-preservation and nutritional competence (SPANC) may be achieved by functional changes to an alternative sigma factor such as RpoS in order to relieve competition for RNA polymerase to bind with the housekeeping sigma factor (σ70) (Ferenci, 2005). In *C. jejuni*, σ54 reportedly contributes to a motility phenotype (Jagannathan et al., 2001) and resistance to some stresses (Hwang et al., 2011).

Neither motility nor a functional stress response is required under our experimental evolution conditions, and both the biosynthesis of flagella and the provision of ATP for of flagellar function are presumably energetically costly for *C. jejuni*. The energy cost associated with flagellar biosynthesis is also implicated in the loss of flagellar gene expression in laboratory evolved *E. coli* (Edwards et al., 2002; Cooper et al., 2003). It is likely that the σ54 loss and the mutations in other genes either involved in flagellar biosynthesis or affecting motility in other ways that we observed all promote faster *C. jejuni* growth through decreased expression of multiple unnecessary flagellar proteins, decreased competition for RNA polymerase binding with the housekeeping sigma factor, σ70, and savings of energy costs required by flagellar function.

In this work we report mutations in *flh*A, *flh*B, *flg*S, *flg*R and *fli*P, genes that affect regulation of the flagellar transcriptional cascade through known regulatory networks. Multiple large genetically irreversible DNA deletions that removed the majority of the *rpo*N reading frame were detected. In some of the deletion events, genes downstream of *rpo*N, a number of which are pseudogenes, were also lost from the genome. Loss of downstream genes was likely collateral to the removal of *rpo*N. Other mutations detected were in tracts of homopolymeric DNA, which are likely subject to a higher mutation rate and are reversible by slip strand mispairing mutations in *C. jejuni* (Parkhill et al., 2000). In our study, one notable homopolymeric tract mutation occurred within a 5 base guanine tract within a gene encoding the flagellar motor protein, *mot*A, and was predicted in every population. This mutation is intriguing considering the hypervariability that occurs in longer (≥ 8 base) homopolymeric guanine tracts in *C. jejuni* (Parkhill et al., 2000; Jerome et al., 2011). Presumably homopolymeric guanine tract mutations occur at a lower rate for shorter tracts, but the fact is that this mutation occurred in all lines suggests that ORF-disrupting indel mutations in short guanine or cytosine tracts are a biologically relevant source of *C. jejuni* genomic diversity. Finally, we show evidence for multiple previously undescribed mutations in long and/or short homopolymeric adenine and thymine tracts in *flg*S, *flg*R, *flh*A, *rpo*N, and *pfl*A that are expected to result in reversible motility loss. Homopolymeric adenine and thymine tracts are ubiquitous in the AT-rich *C. jejuni* genome, and our data are consistent with other work suggesting these tracts are a substantial source of genomic diversity (Hendrixson, 2006), and therefore contribute to evolvability.

The biological significance of reversible motility loss through homopolymeric adenine tract mutations for *C. jejuni* pathogenesis has not been determined. The reversibly non-motile *C. jejuni* isolate (11168mot-) in this work was either cleared from the mouse GI tract before it could establish infection in the C57BL/6 IL-10^–/–^ mouse model or colonized to significantly lower levels than wild-type (11168wt) and the revertant 11168mot+. Although the mutation in 11168mot- was readily reversible on semi-solid agar, *in vivo* restoration of motility occurred at a low rate such that we only detected *C. jejuni* in 2 of 10 11168mot- inoculated mice. We conclude that even reversible motility loss is detrimental to *C. jejuni* fitness in this non-avian animal model of infection. In contrast, Hendrixson showed that a reversibly non-motile isolate was able to consistently colonize the chicken GI tract, but only to low loads unless reversion to the motile phenotype was selected *in vivo* (Hendrixson, 2006). It is possible that the avian intestine, where *C. jejuni* is considered a commensal organism (Young et al., 2007), is more permissive to colonization by motility defective cells than the mouse GI tract and provides a reservoir for the expansion of mutational diversity and increased colonization capacity in subsequent hosts by selection for motility. Within an avian host, *C. jejuni* may also be more exposed to attack by phage that attach to flagella (Coward et al., 2006), so that reversible flagella expression could act as a mechanism for phage avoidance.

We also report here that the restoration of motility *in vitro* did not completely restore the degree of spreading on semi-solid agar, or the ability to colonize mice. The incomplete complementation of the phenotype, despite complementation of the *fli*R frameshift mutation, suggests that subtle genomic differences in 11168mot- also contributed to the motility and colonization deficiency. Indeed, we discovered a non-synonymous single base substitution in *rrc*, which encodes a non-heme iron-binding protein, that was present in the motility mutant (11168mot-) and the revertant (11168mot +), but not wild-type (11168wt). In this case, the modified amino acid is not conserved or part of any known functional motif in homologous proteins from other bacterial species (Yamasaki et al., 2004), but the phenotypic effect of this change, if any, is unknown. We also found differences in homopolymeric guanine tract lengths in contingency loci between 11168wt, 11168mot-, and 11168mot+, and these genes have been shown to be important for *C. jejuni* pathogenicity in this model (Jerome et al., 2011). Genomic differences in these variants evolved from the same stock even though there was minimal laboratory culture. Thus, this type of genetic and phenotypic heterogeneity in stock cultures appears to be a near-inevitable consequence of the hypermutability of the *C. jejuni* genome.

In order to persist, organisms must maintain a balance between genome stability (genome robustness) and the ability to adapt via mutation followed by natural selection (genome evolvability (Lenski et al., 2006). The rapid accumulation of reversible mutations in *C. jejuni* likely increases evolvability and, in contrast to irreversible mutations, may actually maintain genomic robustness since some cells within a population are always one high-rate mutation away from restoration of the ancestral phenotype. The pattern of evolution we observed was characterized by this type of reversible loss of function, before an irreversible loss of function occurred later. This suggests that one mechanism used by *C. jejuni* to attain high evolvability without sacrificing robustness is through high-rate reversible mutations that allow a rapid exploration of the fitness landscape, before function loss under prolonged selective pressure. Here we observed both reversible and irreversible loss-of-function mutations in motility, with irreversible mutations in a major regulatory element coming to dominate the population. This irreversible pattern was not observed in mutations detected in other virulence-related loci that may contribute to growth in broth, for example, the genes involved in iron metabolism. A study on motility defects in *C. jejuni* has shown that the outcome of a deletion of one gene (*fla*B) can be affected by second-site mutations in other genes (*fli*W, and *flg*D) associated with the same phenotype (de Vries et al., 2015). Based on recent studies, genes from the flagellar regulatory cascade were found essential for the fitness of *C. jejuni* 11168 during an *in vitro* growth experiment; *fli*F (MS ring); *fli*G (C-ring), *fli*L (motor); *flg*I and *flg*A (P-ring); *flg*B, *flg*C, *flg*G and *fli*E (rod); *fli*H and *fli*Q (T3SS); and *flg*P and *prf*B_flg (unknown function) (de Vries et al., 2017; Mandal et al., 2017). Interestingly, although these genes are required for fitness in colonization of two different hosts, survival in a third host, and invasion of tissue culture cells, no mutations were detected in any of these genes in our experiment.

In this study, we applied an experimental evolution approach to identify both phenotypic and genotypic changes in a pathogenic *C. jejuni* strain during adaptation in nutrient-enriched medium. This study has advanced understanding of *C. jejuni* adaptability through genomic modification, particularly disruption of the flagellar synthesis cascade following large-scale deletion of the regulatory gene *rpo*N (σ54) and downstream ORFs. Genome reduction is one of the major mechanisms of evolution among prokaryotes; it has been observed to occur gradually as a means to attain maximum growth rate and high adaptability during evolution (Wolf and Koonin, 2013). Our study shows that the percentage of *C. jejuni* mutants with deletion of *rpo*N and downstream genes increased over time, and evolved *C. jejuni* populations exhibited increased growth rates. We expect that a longer-term study of this design would lead to erosion of other genes that are non-essential for *C. jejuni* in this environment. In addition, if environmental changes were made in the *in vitro* environment to make a particular *rpo*N-dependent abiotic stress response essential (Hwang et al., 2011), *rpo*N deletion mutants could be used to determine whether new regulatory mechanisms could evolve to preserve those genes and their functions. Thus, such studies could elucidate mechanisms of the evolution of genetic regulation.

## Conclusion

This study demonstrates one of the mechanisms underlying rapid adaption to a novel laboratory environment by an important enteric pathogen of humans and animals. Previously, we showed that standing genetic variation in contingency loci drove the rapid adaptation of *C. jejuni* to a mouse model host resulting in increased virulence. We determined that *C. jejuni* exists as a population of genotypes within a host, acting as a quasi-species. Data showed that some contingency loci phases sweep to near fixation during passage, while others were stable as a mixed population. Selection at one locus did not have any effect on the distribution of mutations at other loci in that previously published *in vivo* study (Jerome et al., 2011). We concluded that in the host *C. jejuni* exists as a population of genotypes and phenotypes, and not a clonal isolate. Conversely, it has been recognized that when *C. jejuni* is passaged repeatedly under laboratory conditions it eventually loses ability to infect animal hosts, yet the full range of underlying genetic changes underlying these changes was largely undescribed. In this study we started with highly virulent, mouse adapted *C. jejuni* 11168 isolates from our previous study and repeatedly passaged them in parallel in rich culture medium in a time course design to examine their evolution under these conditions. This pathogenic *C. jejuni* strain was rapidly attenuated by experimental laboratory evolution and demonstrated genomic instability during this evolutionary process. Population re-sequencing revealed numerous disruptive mutations to genes in the flagellar transcriptional cascade, including genes (*flg*R, *flg*S, *flh*A, *flh*B) affecting the expression of *rpo*N (σ54) and therefore the expression of the genes σ54 regulates, coupled with deletion of *rpo*N in all evolved lines. Loss of motility in evolved populations was driven by phase variation, slip-strand mutations, and gene loss. The changes observed suggest *C. jejuni* is able to evolve in a novel environment through genome reduction as well as transition, transversion, and slip-strand mutations. These findings advance knowledge about genomic potential of *C. jejuni* and evolutionary mechanisms to adapt to a new environment.

## Data Availability Statement

The datasets presented in this study can be found in online repositories. The names of the repository/repositories and accession number(s) can be found in the article/Supplementary Material.

## Ethics Statement

All mouse experiments were performed according to recommendations in the Guide for the Care and Use of Laboratory Animals of the National Institutes of Health. Protocols were reviewed and approved by the Michigan State University Institutional Animal Use and Care Committee (approval numbers 06/12-107-00 and 06/15-101-00).

## Author Contributions

JPJ, JAB, and LSM conceived the project. LSM wrote the grant and achieved funding for the project. JPJ, JAB, and LSM designed the study and performed mouse experiments. JPJ, AAS, JY, and HYK performed the evolutionary analyses and motility assays. JPJ and AAS performed the DNA isolation and sequencing analyses. JEB trained JPJ and AAS for bioinformatics analyses. JPJ, AAS, and JAB analyzed the sequencing data. JPJ, AAS, JAB, and LSM analyzed the data, performed statistical analyses, and wrote and drafted the manuscript. All authors discussed the data and contributed to the completion of the final manuscript. JAB and LSM edited the manuscript. JAB and LSM supervised the study.

## Conflict of Interest

The authors declare that the research was conducted in the absence of any commercial or financial relationships that could be construed as a potential conflict of interest.

## Footnotes

1 http://barricklab.org/breseq
