# Supplementary figures and images for: Experimental Evolution of Campylobacter jejuni Leads to Loss of Motility, rpoN (σ54) Deletion and Genome Reduction
Source: Front Microbiol. 2020 Nov 6;11:579989. doi: 10.3389/fmicb.2020.579989 (PMC7677240; doi:10.3389/fmicb.2020.579989)

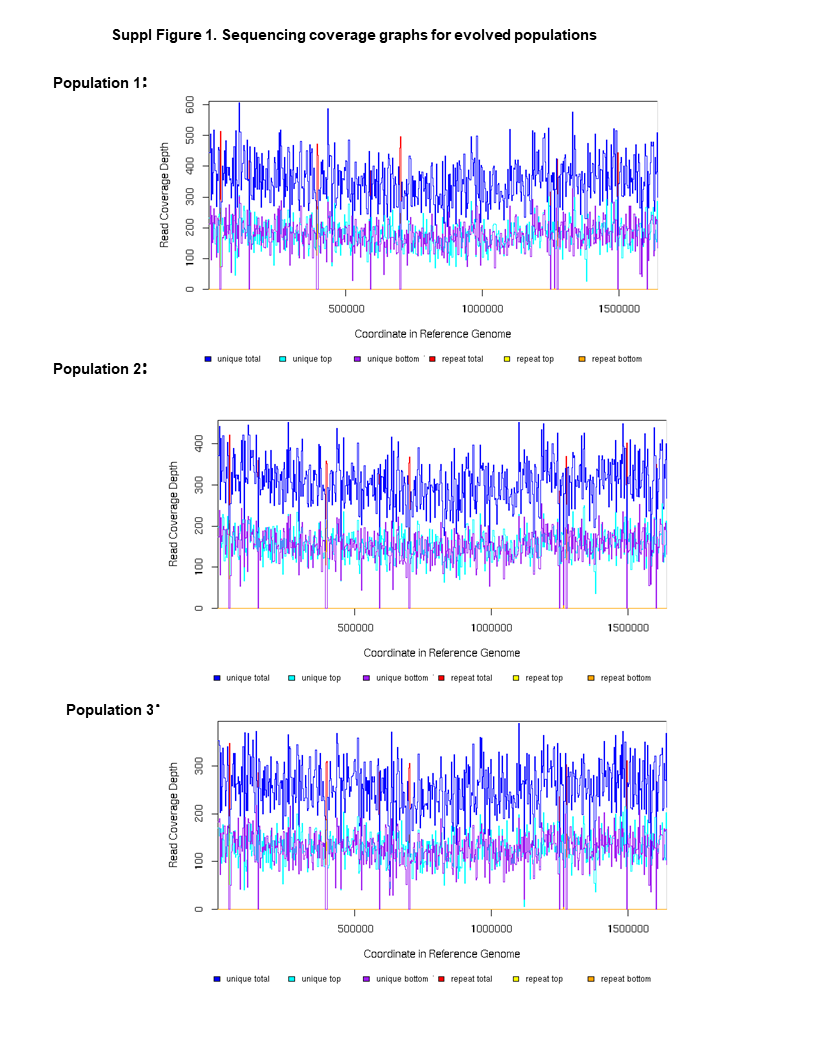

Supplement: Supplementary file 1 [file Image_1.TIF]

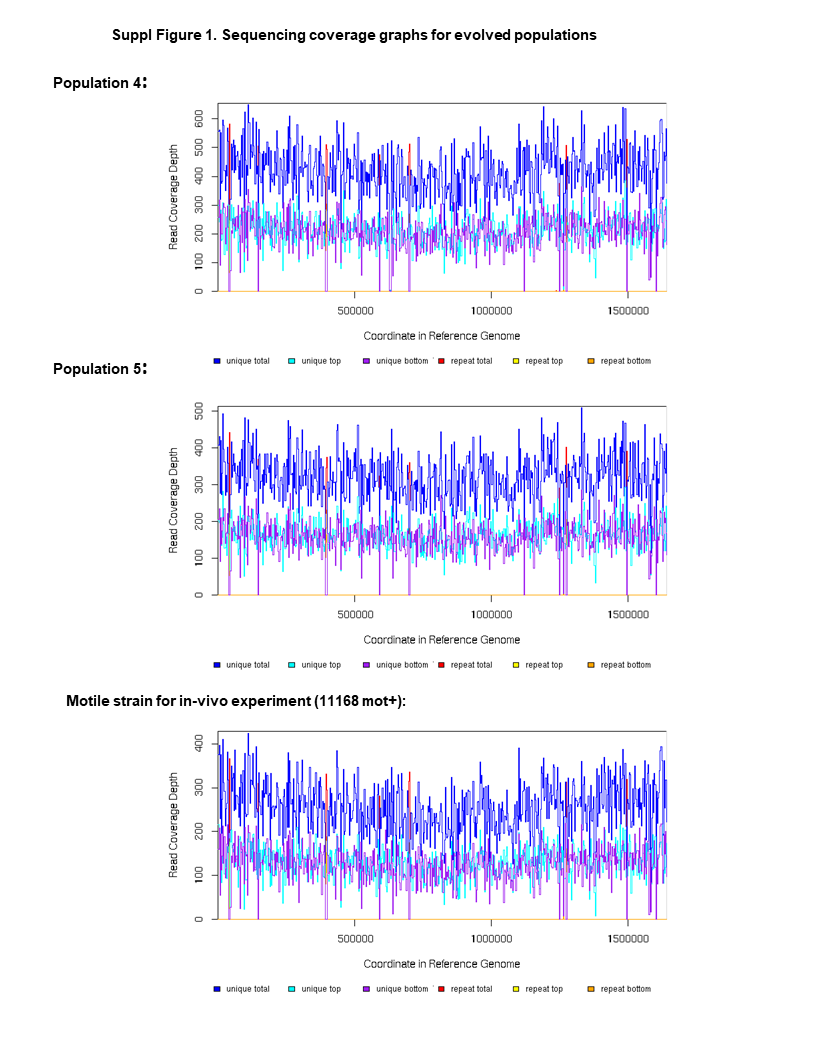

Supplement: Supplementary file 2 [file Image_2.TIF]

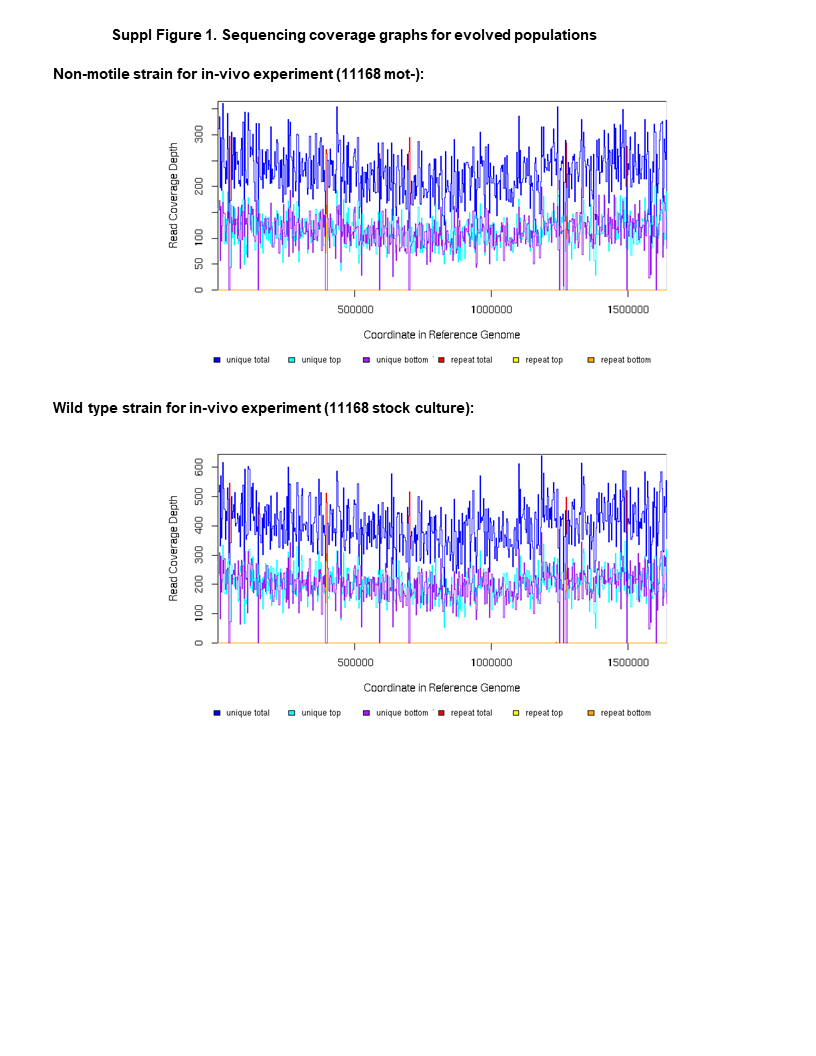

Supplement: Supplementary file 3 [file Image_3.TIF]
